# Clinical Correlation of Altered Molecular Signatures in Epileptic Human Hippocampus and Amygdala

**DOI:** 10.1007/s12035-023-03583-6

**Published:** 2023-09-02

**Authors:** Sayed Mostafa Modarres Mousavi, Fatemeh Alipour, Farshid Noorbakhsh, Maryam Jafarian, Masoud Ghadipasha, Jaber Gharehdaghi, Christoph Kellinghaus, Erwin-Josef Speckmann, Walter Stummer, Maryam Khaleghi Ghadiri, Ali Gorji

**Affiliations:** 1 Shefa Neuroscience Research Center, Khatam Alanbia Hospital, Tehran, Iran; 2 Legal Medicine Research Center, Legal Medicine Organization, Tehran, Iran; 3 Department of Neurology, Klinikum Osnabrück, Osnabrück, Germany; 4 Department of Neurosurgery, Westfälische Wilhelms-Universität Münster, Münster, Germany; 5 Epilepsy Research Center, Westfälische Wilhelms-Universität Münster, Münster, Germany; 6 Department of Neuroscience, Mashhad University of Medical Sciences, Mashhad, Iran

**Keywords:** Intractable epilepsy, Brain, Receptors, Ion channels, Epileptogenesis

## Abstract

Widespread alterations in the expression of various genes could contribute to the pathogenesis of epilepsy. The expression levels of various genes, including major inhibitory and excitatory receptors, ion channels, cell type-specific markers, and excitatory amino acid transporters, were assessed and compared between the human epileptic hippocampus and amygdala, and findings from autopsy controls. Moreover, the potential correlation between molecular alterations in epileptic brain tissues and the clinical characteristics of patients undergoing epilepsy surgery was evaluated. Our findings revealed significant and complex changes in the expression of several key regulatory genes in both the hippocampus and amygdala of patients with intractable epilepsy. The expression changes in various genes differed considerably between the epileptic hippocampus and amygdala. Different correlation patterns were observed between changes in gene expression and clinical characteristics, depending on whether the patients were considered as a whole or were subdivided. Altered molecular signatures in different groups of epileptic patients, defined within a given category, could be viewed as diagnostic biomarkers. Distinct patterns of molecular changes that distinguish these groups from each other appear to be associated with epilepsy-specific functional consequences.

## Introduction

Approximately 30% of patients with mesial temporal lobe epilepsy (MTLE), the most frequent form of recurrent unprovoked focal seizures, are refractory to anticonvulsant therapy [1–3]. The mechanisms underlying MTLE remain poorly understood. Although several investigations in different animal models indicate contributions of various mechanisms to the development of seizures, the corresponding evidence from the human epileptic brain is scarce [4]. Experimental and clinical investigations indicate that a wide range of mechanisms can contribute to seizure genesis in MLTE, including changes in receptors and neurotransmitters [5], dysregulation of ion channels and neuropeptide signaling pathways [6], alterations in interneuronal circuits [7], neural network reorganization [8], angiogenesis and blood-brain-barrier dysfunction [9], gliosis [10], and cellular damage/death [11]. Numerous studies have revealed that the alterations of both excitatory and inhibitory receptors in various brain regions of the epileptic human brain contribute to the onset and spread of seizures [12, 13]. Dysfunction of glutamate and/or GABA synaptic neurotransmissions in different brain regions plays pivotal roles in disrupting the excitatory/inhibitory synaptic balance and triggering epileptic discharges [11, 14]. Many investigations provided detailed data on the expression of different receptors and putative changes in the subunit composition of various receptors in the human epileptic hippocampus [15–17], amygdala [18, 19], and neocortex [20, 21]. Furthermore, it has been shown that alterations of synaptic neurotransmission in epileptic neuronal tissues contribute to histopathological changes, such as cell injury/death and gliosis in MTLE [11]. In addition to receptors and ion channels, dysregulation of glial functions, such as malfunction and/or downregulation of glutamate transporters, is involved in the generation of epileptic seizures and pathological changes of MTLE [22, 23]. Both experimental and clinical investigations suggest that several factors, such as the frequency [24] and intensity [25] of seizures, prolonged seizures [26], anticonvulsants [27, 28], age of onset [29, 30], and psychiatric/cognitive abnormalities [31], could affect the histological and molecular architecture of the epileptic brain. The expression levels of various genes of both the human epileptic hippocampus and amygdala were investigated. We aimed to (i) explore the expression of major inhibitory and excitatory receptors as well as ion channels, cell type-specific markers, and excitatory amino acid transporters in the hippocampus and the amygdala, which were systematically compared to findings from autopsy controls, and (ii) to evaluate the potential correlation between the molecular alterations of the epileptic brain tissues and demographic as well as clinical characteristics of patients undergoing epilepsy surgery.

## Materials and Methods

### Tissue Collection

All experiments were conducted following the National Institute of Health Guide for Care and Use and were approved by the Ethics Committee of Shefa Neuroscience Research Center, Tehran, Iran. Informed consent was obtained from all patients. The study included individuals who met the following criteria: (i) patients aged 12 years or older with medically intractable epilepsy, (ii) patients without any systemic disease, and (iii) patients without a history of status epilepticus. After undergoing trials of at least two well-tolerated and appropriately selected anticonvulsants (at adequate doses), these patients have shown resistance to medication therapy [32]. All patients had undergone pre-surgical assessments, and surgical intervention was suggested to achieve seizure control. All patients showed unilateral hippocampal sclerosis in MRI and had undergone temporal lobectomy. The brain specimens obtained were a portion of the tissue that had been surgically resected for the treatment of medically refractory focal epilepsy. The tissues were obtained from the hippocampus and amygdala of 19 patients with refractory MTLE (mean age: 33.2 ± 2.6 years) during surgical treatment at Khatam Hospital, Tehran, Iran, between 2011 and 2018. Table 1 provides detailed patient characteristics, including gender, age, age of seizure onset, epilepsy duration and frequency, the estimated total number of seizures, antiepileptic drugs (AEDs) used, surgical outcomes, presence of associated psychiatric disorders, magnetic resonance imaging findings, dominant hemisphere, and major historical risk factors for developing epilepsy (such as brain trauma and febrile seizure). As control tissue, the hippocampus and amygdala were obtained during autopsies performed on bodies from the body donor program of the Forensic Medicine Organization, Tehran, Iran. The control subjects (*n* = 14; mean age: 42.6 ± 3.5 years) had no known medical history of any neurological and psychiatric diseases. The causes of death were cardiac arrest (*n* = 6), cardiorespiratory insufficiency (*n* = 3), abdominal trauma (*n* = 3), multiple organ failure (*n* = 1), and acute respiratory infection (*n* = 1, Table 2). Autopsy delay varied between 2 and 8 h.

**Table 1:** Clinical history of patients

No	Gender	Age (year)	Age of seizure onset (year)	Epilepsy duration (year)	Seizure frequency (number of seizure)	Estimated total number of seizures	Drugs	ILAE	Psychiatric disorders	MRI	Dominant lobe	Risk factor of epilepsy
1	Male	35	10	25	Weekly (4)	5200	CBZ, CLZP	2	No	Left sclerosis	Yes	None
2	Male	47	17	30	Weekly (3)	4680	PHB, PHT, CBZ	1	No	Right sclerosis	No	Trauma
3	Female	27	11	16	Weekly (3)	2496	LTG, CBZ, LEV	1	No	Left sclerosis	Yes	None
4	Male	23	13	10	Daily (1)	3650	CBZ, LEV, VPA	6	No	Left sclerosis	Yes	None
5	Female	52	7	45	Monthly (1)	540	LEV, CBZ	1	Yes	Left sclerosis	Yes	None
6	Male	48	28	20	Monthly (1)	240	LEV, CBZ, PHT, LTG	1	No	Left sclerosis	Yes	Trauma
7	Female	17	5	12	Monthly (10)	1440	VPA, CBZ	1	Yes	Left sclerosis/dysplasia	Yes	None
8	Male	18	14	4	Daily (5)	7300	CBZ, TPM	1	No	Left sclerosis	Yes	None
9	Male	28	8	20	Weekly (2)	2080	CBZ, TPM, VPA	1	No	Right sclerosis	No	Febrile seizure
10	Male	34	3	31	Weekly (2)	3224	CBZ	1	No	Left sclerosis	Yes	Trauma
11	Female	30	18	12	Weekly (2)	1248	LEV, TPM, CBZ, LTG	1	Yes	Right sclerosis	Yes	None
12	Female	24	1	23	Weekly (10)	11,960	LTG, LEV	1	Yes	Right sclerosis	No	None
13	Female	39	4	35	Monthly (3)	1260	CBZ, LTG, LEV	1	Yes	Right sclerosis	No	None
14	Male	35	32	3	Monthly (2)	72	CBZ, VPA	1	Yes	Right sclerosis	No	None
15	Male	30	4	26	Daily (10)	94,900	CBZ, TPM, VPA, LEV	1	Yes	Right Sclerosis	Yes	Trauma
16	Male	50	18	32	Daily (10)	116,800	CBZ, TPM, PHT, PHB, VPA, PRM	1	Yes	Left sclerosis/dysplasia	Yes	None
17	Female	37	7	30	Daily (2)	21,900	LTG, CBZ, PRM	1	Yes	Left sclerosis	Yes	Febrile seizure
18	Male	43	19	24	Weekly (1)	1248	OCBZ, LEV	1	No	Left sclerosis	Yes	Febrile seizure
19	Male	14	1.5	12.5	Weekly (1)	650	VPA, LEV, OCBZ, PRM	3	No	Left sclerosis	Yes	Trauma

*CBZ*, carbamazepine; *CLZP*, clonazepam; *PHB*, phenobarbital; *PHT*, phenytoin; *LTG*, lamotrigine; *LEV*, levetiracetam; *VPA*, valproate; *TPM*, topiramate; *PRM*, pyrimidone; *OCBZ*, oxcarbazepine; *MRI*, magnetic resonance imaging

**Table 2:** Etiology of autopsy control

No	Age (year)	Etiology
1	57	Cardiorespiratory insufficiency
2	30	Cardiorespiratory insufficiency
3	30	Acute respiratory infection
4	53	Cardiac arrest
5	47	Cardiac arrest
6	45	Abdominal trauma
7	38	Cardiac arrest
8	48	Abdominal trauma
9	68	Multiple organ failure
10	50	Cardiac arrest
11	39	Cardiorespiratory insufficiency
12	30	Cardiac arrest
13	44	Cardiac arrest
14	18	Abdominal trauma

### Extraction and Reverse Transcription

Total RNA was isolated from the epileptic hippocampus and amygdala specimens, as well as the autoptic hippocampus and amygdala tissues (control), using RNeasy® lipid tissue Mini Kit (Qiagen, Germany). A DNase treatment was carried out to remove genomic DNA by RNase-Free DNase Set “DNase I” (Qiagen, Germany). A total RNA amount of 500 ng was used for reverse transcription into cDNA by PrimeScript^TM^ RT reagent kit (Takara, China) according to the manufacturer’s instructions. Primer sequences for β-actin [33], GAPDH [34], and HPRT [35] were designed based on the previous studies. The specific primers were designed using AlleleID®7.50 (Premier Biosoft International, USA). The primer pairs used in this study were either spanning exon-exon junctions or located on different exons. The best primer concentration for a given assay was determined. The efficiency of qRT-PCR was two for each pair of primers and was calculated using serial 1:2 dilutions of template cDNA on a CFX 96 Real-Time System (Bio-Rad, Germany). The primer sequence data are presented in Table 3.

**Table 3:** Primer sequences of neurotransmitters

Biomarkers	Forward primer 5' to 3'	Reverse primer 5' to 3'	Amplicon length (bp)	Ta (°C)
GABA_A_Rα1	GACTGGAAGAAGCTATGGACAG	GTCCGAAACTGGTGACGAAG	173	60.5
GABA_A_Rβ3	TGCTGTATGGGCTCAGAATC	CCTTTCCACTCCGGTAACAG	170	62.7
GABA_A_Rγ2	ACCACCGAAGTAGTGAAGAC	GGACAGTGGTGATACCTAAAGA	196	59
GABA_C_Rp2	CTCTGGGTCAGCTTTGTGTT	TTGGCCTCAGACTCACTGTA	179	62.8
GABA_B_R1	TACGGTTCCATGTTCACCAA	GCAAATGTCTCAATGGTCCG	194	62.7
GABA_B_R2	GGGACTTCTCATGTTGTTCG	ATGATCCCCACGTTGTAGAC	114	60.1
GAD_65_	TATTTTCTCCCGGTGGCG	TCACGCTGTCTGTTCCAATC	186	60.5
NR1	ACCAGACTGAAGATTGTGACG	CAAAAGCCGTAGCAACACTG	191	64
NR2B	GGGAGGAATTTGTCCTTCAGTG	TTTATTCTCAGTGACTATGCGTTTT	300	62
GluR1	TTTGAGGGCAATGACCGTTA	ACATCTGCTCTTCCATAGACC	179	62.7
GluR2	GGAACGGCGTGTAATTCTGGAC	AGGTCTCCATCAGTAAATCCCAGA	132	62.7
mGluR1α	GAATGTCCGCAGTGCCTTCA	CCATTAGAATTGGCATTCCCTGC	138	63.5
Cav2.1	CACCGTCGTACAAGTGAACA	TCTCAAAGTAGCGCAGGTTC	200	62.2
LGI1	AGACATACTTTCCGGGGACT	ATTACCCCTCAGGTCCACAT	126	61
SCN1α	AGAAATGAGACTGCTCGATGGAA	CTTAGGCTGGAGTTCCACATTT	153	59.5
SCN1ß	CCGTGTATGGGATGACCTTC	CATAGCGCAGGATCTTGACA	132	62
SCN2α	CAGCAGCGAGTCAGATATGGA	CTTCCGTACACAGTCTTCTGTAA	169	62.2
GFAP	ATCCACGAGGAGGAGGTTCG	CATACTGCGTGCGGATCTCT	121	64.5
NeuN	GGATGGATTTTATGGTGCTGAG	GTAGACTCTGCCGTAACTGTC	106	61
EAAT1	CCAGCAGGGAGTCCGTAAAC	AGCAGCACAAAAGCATTCCG	105	58.5
EAAT2	AGATGAATGCAAGGTAACTCTGG	CATAGGATACGCTGGGGAGT	135	59.5
NLE1	CAGCCCTACGGGAAAGTACC	GCCATCTGGAGACCAGGATA	139	62
β-actin	AGGCGGACTATGACTTAGTTGCGTTACACC	AAGTCCTCGGCCACATTGTGAACTTTG	220	62
Gapdh	AATCCCATCACCATCTTCCA	TGGACTCCACGACGTACTCA	82	60
HPRT	GGACTAATTATGGACAGGACTG	GCTCTTCAGTCTGATAAAATCTAC	195	61

### Real-time PCR

Real-time PCR reactions were carried out in duplicate on a CFX 96 Real-Time System (Bio-Rad, Germany). EvaGreen dye (Solis BioDyne, Estonia) was used to detect amplified products. PCR amplifications were carried out in a total volume of 20 μl containing 2 μl template, 0.3 μl forward primer and 0.3 μl reverse primer (10 μM; Macrogen, The Netherlands), and 4 μl of 5x HOT FIREPol® EvaGreen® qPCR Mix Plus and 13.4 μl RNase/DNase-free sterile water (Sigma, Germany). The thermocycling conditions consisted of an initial 15 min denaturation condition at 95 °C, then 45 cycles at 95 °C for 30 s, primer annealing at Ta for 30 s, and elongation at 72 °C for 30 s. Following PCR, a melting curve analysis was performed to determine product specificity.

The expression of inhibitory GABA_A_ (Rα1, Rβ3, and Rγ2), GABA_B_ (R1 and R2), GABA_C_ (Rp2) subunits, and GAD_65_ as well as excitatory glutamate subreceptors NMDA (NR1, NR2B), AMPA (GluR1 and GluR2), and mGluR1α subunits, sodium voltage-gated channel (SCN1α and SCN1β), the P/Q voltage-dependent calcium channel (Cav2.1), glioma inactivated protein 1 (Leucine-rich glioma inactivated 1, LGI1), glial fibrillary acidic protein (GFAP), neuronal nuclei (NeuN), notchless protein homolog 1 (NLE1), and excitatory amino acid transporters (EAAT1 and EAAT2) were determined for both autoptic control tissues and epileptic hippocampus and amygdala specimens.

### Statistical Analyses

All statistical analyses were performed using SPSS version 22 software. To compare gene expression levels between the control and epileptic tissues, an independent t-test or Mann-Whitney U test was used for data that were or were not normally distributed, respectively. When comparing gene expression levels between more than two groups, ANOVA followed by Tukey post-hoc was used for normally distributed data. For data that deviated from normality, the Kruskal-Wallis test was performed. In all cases, the Shapiro-Wilk test was carried out to check for data normality. To explore whether gene expression levels might be used in classifying samples into different groups, the receiver operating characteristic (ROC) curve analyses were performed on gene expression data. ROC curve analyses were limited to genes, which had shown significant differences between different groups (based on mean comparison testing). For genes that demonstrated significant “total area under curve” (AUC) values, Youden’s J point was calculated to determine the best “cut-off” value, which maximized sensitivity plus specificity. To explore the existence of correlations between different parameters, Pearson’s *r* or Spearman’s rho correlation coefficients were calculated for the data with and without normal distribution, respectively. These correlation analyses were performed in a pairwise manner between gene expression levels and clinical variables (i.e., between expression levels of one gene as the explanatory variable and one clinical feature as the outcome variable). To evaluate the effects of multiple variables and correct for the effects of other variables, we then performed a set of multiple regression analyses. For these analyses, expression levels of all of the genes were entered into the software as (potential) explanatory variables with each clinical feature as the outcome variable. These analyses were performed to determine whether the relative fold change of different genes might be able to predict the estimated number of seizures, age of seizure onset, and epilepsy duration. For all statistical analyses, *p*-values below 0.05 were considered statistically significant.

## Results

**Fig. 1:**
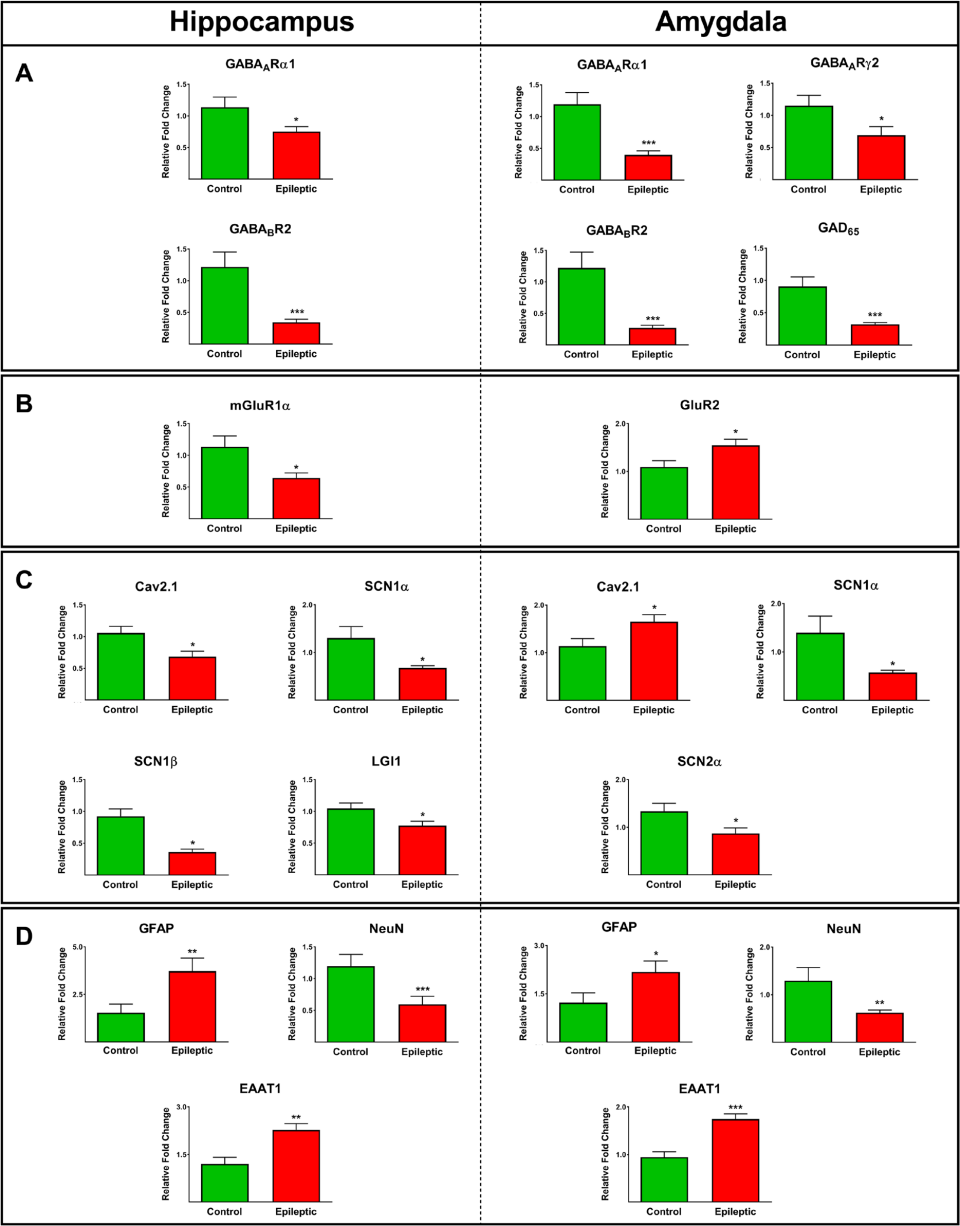
The expression of various genes in the hippocampus and amygdala of epileptic patients and autopsy controls. The expression levels of various GABA receptors and GAD65 (A), glutamate receptors (B), ion channels, and glioma-inactivated 1 gene (LGI1) (C), as well as GFAP NeuN and EAAT1 (**D**), in the epileptic hippocampus and amygdala were significantly different than those in autopsy controls. *, **, and *** indicate *P* ≤ 0.05, *P* ≤ 0.01, and *P* ≤ 0.001, respectively.

The gene expression values of different GABA_A_, GABA_B_, and GABA_C_ receptor subunits, as well as GAD65, and subreceptors of glutamate NMDA and AMPA subunits were assessed in the hippocampus and amygdala of 19 epileptic patients. These findings were then compared to gene expression levels in control autoptic tissues. The expression levels of GABA_A_Rα1 and GABA_B_R2 were significantly lower in the epileptic hippocampus and amygdala compared to the control autoptic tissues (*P* ≤ 0.05; Fig. 1A). Furthermore, the expression values of GABA_A_Rγ2 (*P* ≤ 0.05), as well as GAD_65_ (*P* ≤ 0.001), were significantly lower in the amygdala of epileptic patients compared to the autoptic samples (Fig. 1A). The mGluR1α gene expression in the epileptic hippocampus was significantly lower than in the control tissues (*P* ≤ 0.05; Fig. 1B), whereas the relative gene expression of AMPA receptor subunit GluR2 in the amygdala was significantly higher in the epileptic amygdala compared with autopsy tissues (*P* ≤ 0.05; Fig. 1B).

Furthermore, the expressions of voltage-dependent calcium channel Cav2.1 as well as voltage-gated sodium channels SCN1α, SCN2α, and SCN1β in the hippocampus and amygdala of epileptic and control tissues were assessed. The relative expression levels of Cav2.1, SCN1α, and SCN1β in the epileptic hippocampus were significantly lower than in the autoptic hippocampus (*P* ≤ 0.05; Fig. 1C). The value of Cav2.1 expression in the epileptic amygdala was significantly higher than in control samples, whereas the expressions of SCN1α and SCN2α channels were lower than in the autoptic amygdala (*P* ≤ 0.05; Fig. 1C). Furthermore, the expression of LGI1, a human-epilepsy related gene, was significantly lower in the epileptic hippocampus compared to the autoptic samples (*P* ≤ 0.05; Fig. 1C).

**Fig. 2:**
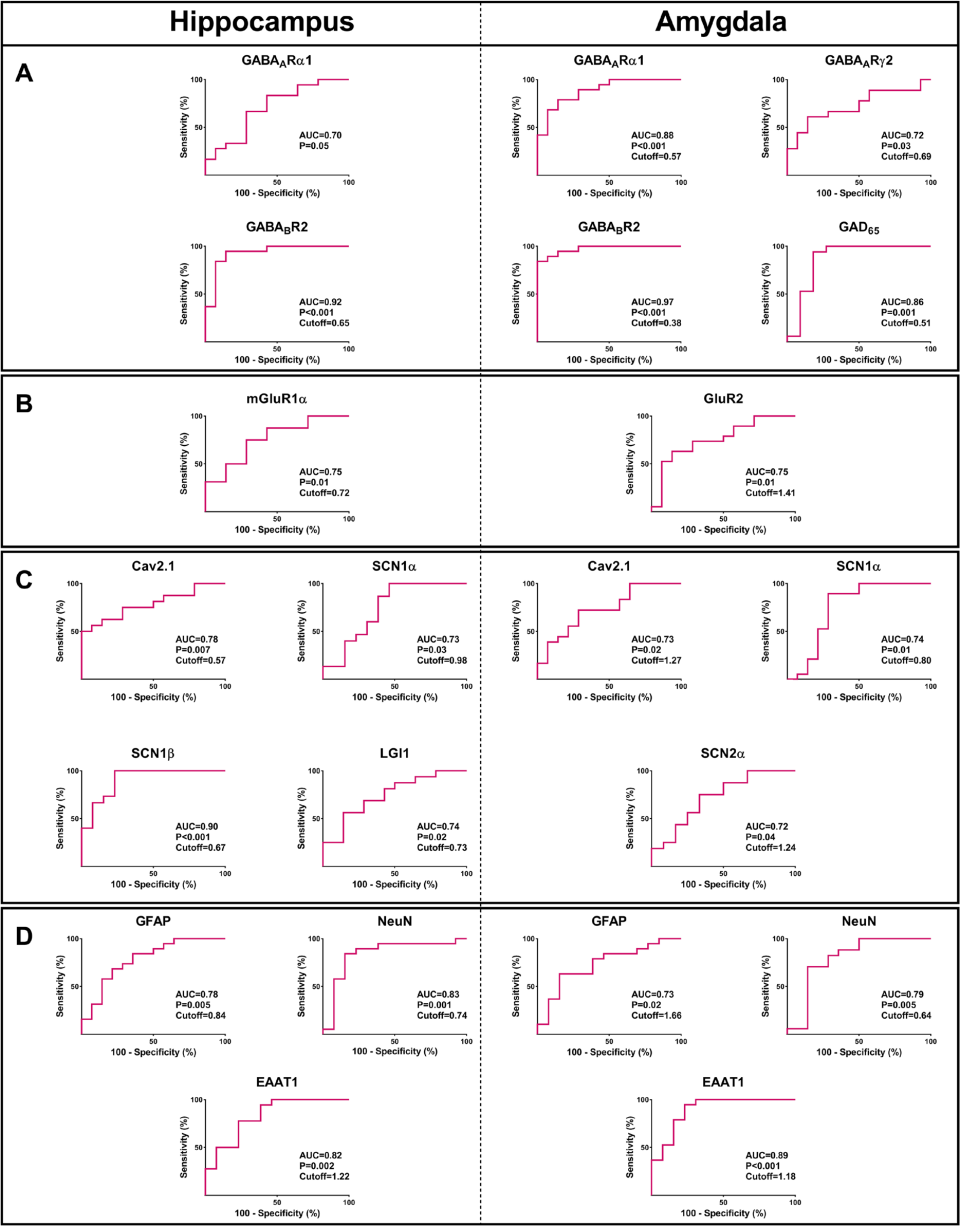
Receiver operating characteristic (ROC) curve analysis assessed differentially expressed genes in the hippocampus and amygdala. The ROC curve of differentially expressed genes was used to distinguish epileptic patients from non-epileptic controls and determine the best cut-off values. The AUCs of tested genes were between 0.7 and 0.97 and were all statistically significant. Cut-off calculated based on Youden’s J statistic test

Moreover, the expressions of glutamate transporters EAAT1 and EAAT2, GFAP, an astrocyte-specific intermediate filament protein, and NeuN, a neuron-specific nuclear protein, were assessed in the epileptic and autoptic specimens. The relative expressions of GFAP and EAAT1 in the epileptic hippocampal and amygdala were significantly greater than in control tissues (*P* ≤ 0.05; Fig. 1D). In both the epileptic hippocampus and amygdala, the expression levels of NeuN were found to be lower compared to the autoptic control tissues (*P* ≤ 0.001; Fig. 1D). Roc curve analysis was employed to evaluate the potential of differentially expressed genes in distinguishing patients with epilepsy from non-epileptic controls. This analysis aimed to identify the optimal cut-off values that could effectively discriminate between the two groups. As is evident in Fig. 2, the AUCs of tested genes were between 0.7 and 0.97 and were all statistically significant.

### Correlation between Epilepsy-associated Gene Expression and Patient Characteristics

**Fig. 3:**
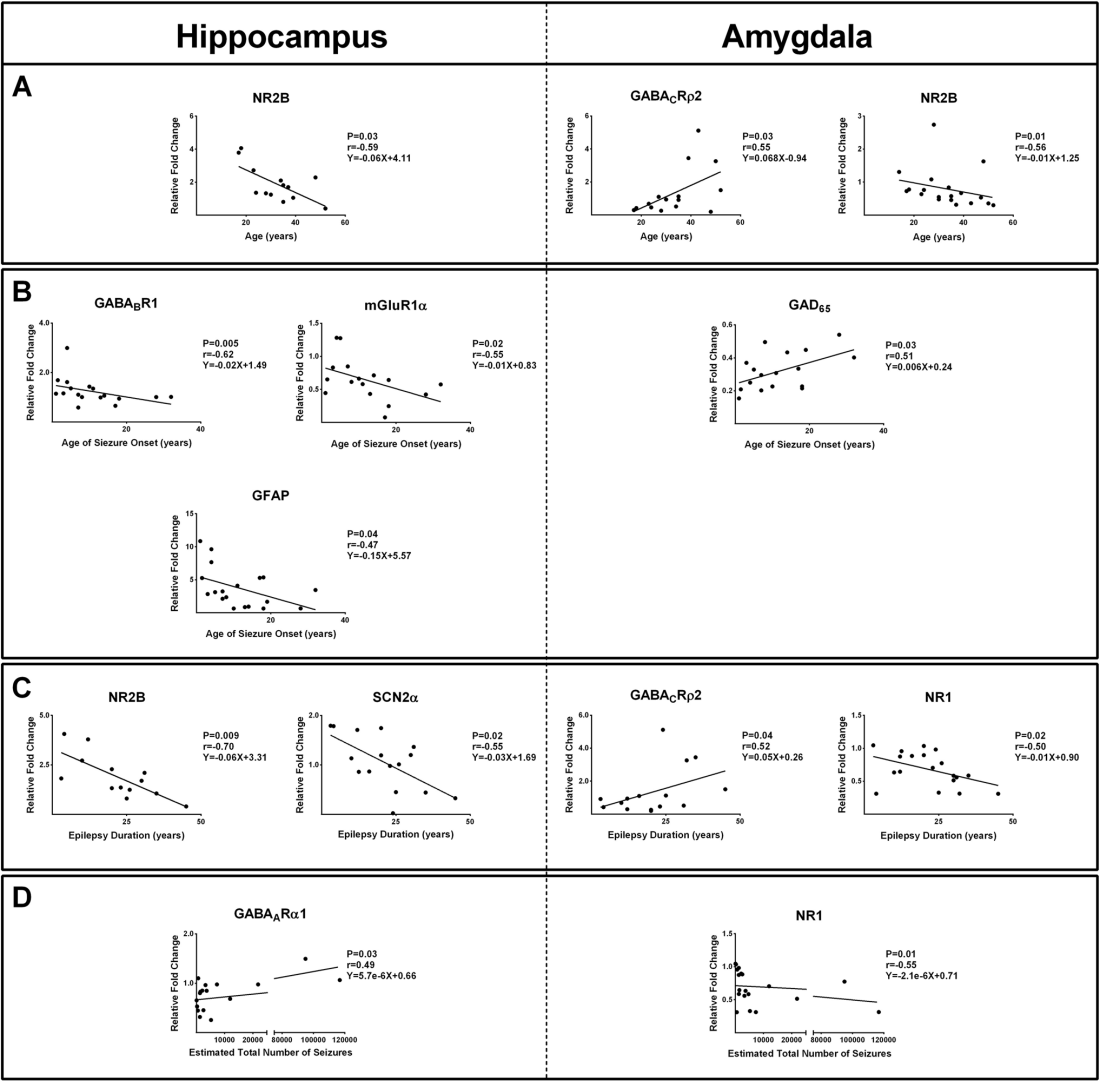
Correlation between various gene expressions and patient characteristics. There was a significant negative or positive correlation between the expression of various genes in the hippocampus and amygdala and the age of patients (A), age of seizure onset (B), epilepsy duration (C), and the estimated total number of seizures (D)

We found a significant negative correlation between the expression of NR2B in the hippocampus and the age of patients (*r* = 0.59; *P* = 0.03; Fig. 3A). Furthermore, significant negative correlations between the expressions of GABA_B_R1 (*r* = 0.62; *P* = 0.005), mGluR1α (*r* = 0.55; *P* = 0.02), and GFAP (*r* = 0.47; *P* = 0.04) in the hippocampus and the age of seizure onset were observed (Fig. 3B). Lower NR2B (*r* = 0.7; *P* = 0.009) and SCN2α (*r* = 0.55; *P*=0.02) levels in the hippocampus were accompanied by an increase in epilepsy duration (Fig. 3C). There was a significant positive correlation between the GABA_A_Rα1 expression in the hippocampus and the estimated total number of seizures (*r* = 0.49; *P* = 0.03; Fig. 3D).

In the amygdala, a significant positive correlation was observed between the value of GABA_C_Rρ2 expression and the age of the patients (*r* = 0.55; *P* = 0.03; Fig. 3A). Moreover, there was a significant negative correlation between the value of NR2B gene expression and the age of the patients (*r* = 0.56; *P* = 0.01; Fig. 3A). The expression of GAD_65_ in the amygdala had a significantly positive correlation with the age of seizure onset (*r* = 0.51; *P* = 0.03; Fig. 3B). Our study indicated the presence of a significant positive correlation between the expression of GABA_C_Rρ2 and the duration of epilepsy (*r* = 0.52; *P* = 0.04). Additionally, a negative correlation was observed between the values of NR1 and the duration of epilepsy (*r* = 0.5; *P* = 0.02; Fig. 3C). Besides, a decrease in NR1 expression in the amygdala was accompanied by an increase in the estimated total number of seizures (*r* = 0.55; *P* = 0.01; Fig. 3D).

### Epilepsy-associated Gene Expression and Psychiatric Disorders

A distinctive characteristic of MTLE is its heterogeneity [36]. Recognizing this inter-individual variability in gene expression among epileptic patients, we performed data subdivision and analyzed the relationships between gene expression and various clinical parameters.

**Fig. 4:**
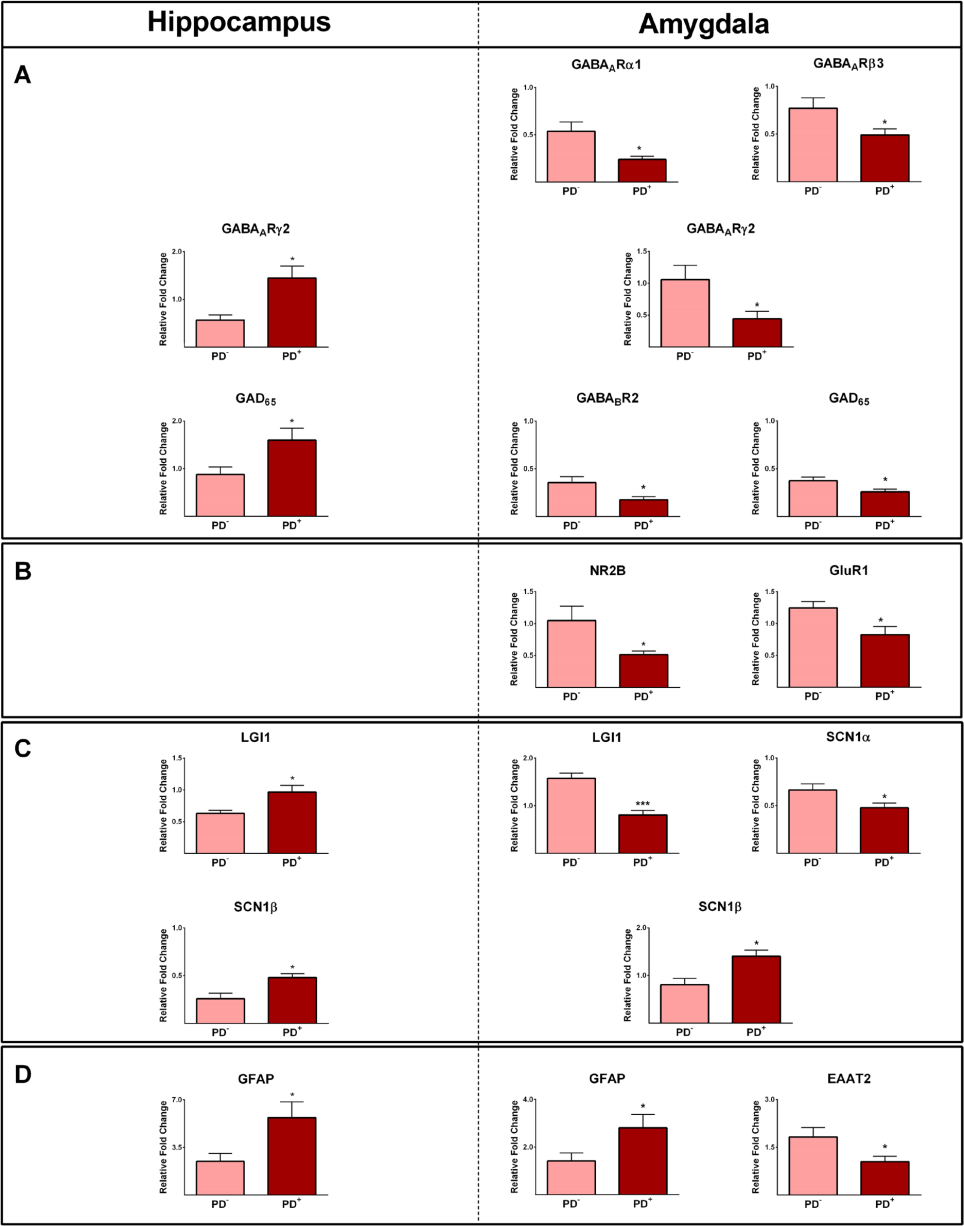
Expression of various genes in the hippocampus and amygdala tissues obtained from epileptic patients with (PD^+^) and without (PD^-^) psychiatric disorders. Significant differences were observed in the expression of various GABA receptors and GAD_65_ (**A**), glutamate receptors (**B**), different voltage-gated sodium channels, and glioma-inactivated 1 gene (LGI1; **C**), as well as GFAP and EAAT2 (**D**), in the hippocampus and amygdala obtained from epileptic patients with and without psychiatric disorders. * and *** indicate *P* ≤ 0.05 and *P* ≤ 0.001, respectively

Among 19 subjects with epilepsy, 9 patients suffered from psychiatric disorders, mostly anxiety and/or depression (Table 1). To identify those genes that could be either affected by or contributed to psychiatric disorders, we compared our data from the epileptic hippocampus and amygdala of patients with and without psychiatric disorders. The expressions of GABA_A_Rγ2, GAD_65_, LGI1, SCN1β, and GFAP were significantly higher in the hippocampus of epileptic subjects with psychiatric disorders compared to patients without psychiatric disorders (*P* ≤ 0.05; Fig. 4A–D).

**Fig. 5:**
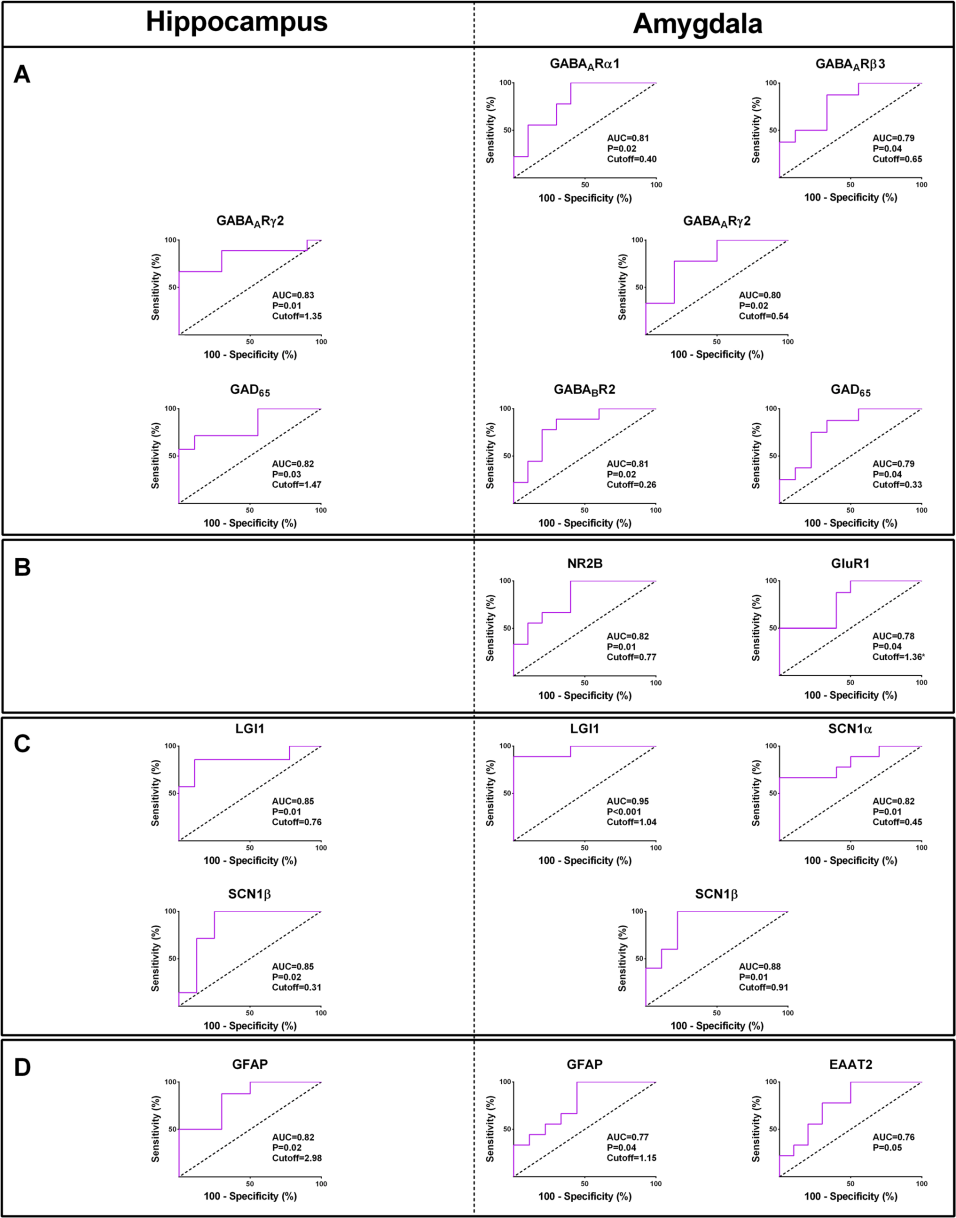
Receiver operating characteristic (ROC) curve analysis was used to assess differentially expressed genes between patients with and without psychiatric disorders and determine the best cut-off values. The AUCs of tested genes were between 0.76 and 0.95 and were all statistically significant. Cut-off calculated based on Youden’s J statistic test

Moreover, the expression values of the GABA_A_Rα1, GABA_A_Rβ3, GABA_A_Rγ2, GABA_B_R2, GAD_65_, NR2B, GluR1, LGI1, SCN1α, and EAAT2 were significantly lower, and the expressions of SCN1β and GFAP were significantly higher in the amygdala of epileptic patients with psychiatric disorders compared to those without psychiatric disorders (*P* ≤ 0.05; Fig. 4A-D). Roc curve analysis was used to assess whether differentially expressed genes could be used to distinguish epileptic patients with psychiatric disorders from those without psychiatric disorders and determine the best cut-off values. Figure 5 depicts the results, revealing that the tested genes exhibited AUC values ranging from 0.76 to 0.95. All of these AUC values were found to be statistically significant.

### Epilepsy-associated Gene Expression and Traumatic Brain Injury (TBI)

**Fig. 6:**
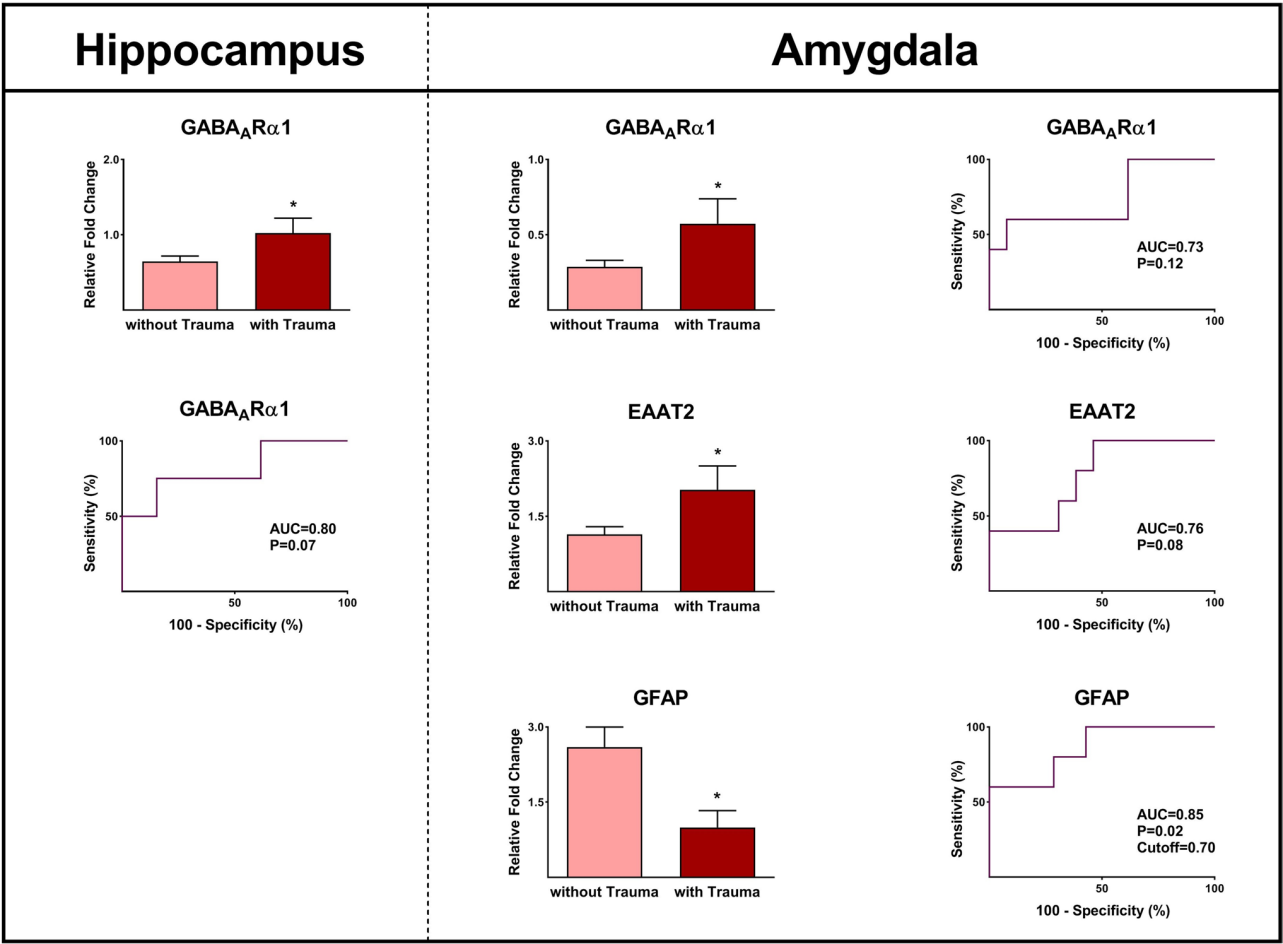
The expressions of various genes in the hippocampus and amygdala tissues were obtained from epileptic patients with and without traumatic brain injury. Significant differences were observed in the gene expression of GABAARα1, EAAT2, and GFAP in epileptic patients with and without traumatic brain injury. The AUCs of tested genes were between 0.73 and 0.85 and were all statistically significant. Cut-off calculated based on Youden’s J statistic test. * indicates *P* ≤ 0.05

Out of the 19 patients included in our study, five of them had a history of moderate-to-severe TBI (Table 1). The hippocampal tissues from epileptic patients with a history of TBI exhibited a significantly higher value of GABA_A_Rα1 compared to those without TBI (*P* ≤ 0.05; Fig. 6). Furthermore, higher values of GABA_A_Rα1 and EAAT2 and lower levels of GFAP were observed in the amygdala of patients with a history of TBI compared with epileptic subjects without TBI (*P* ≤ 0.05; Fig. 6). Based on the ROC curve analysis displayed in Fig. 6, it can be observed that the AUC values of genes with differences between the two groups ranged from 0.73 to 0.85. These AUC values were found to be statistically significant.

### Epilepsy-associated Gene Expression and Age of Seizure Onset

**Fig. 7:**
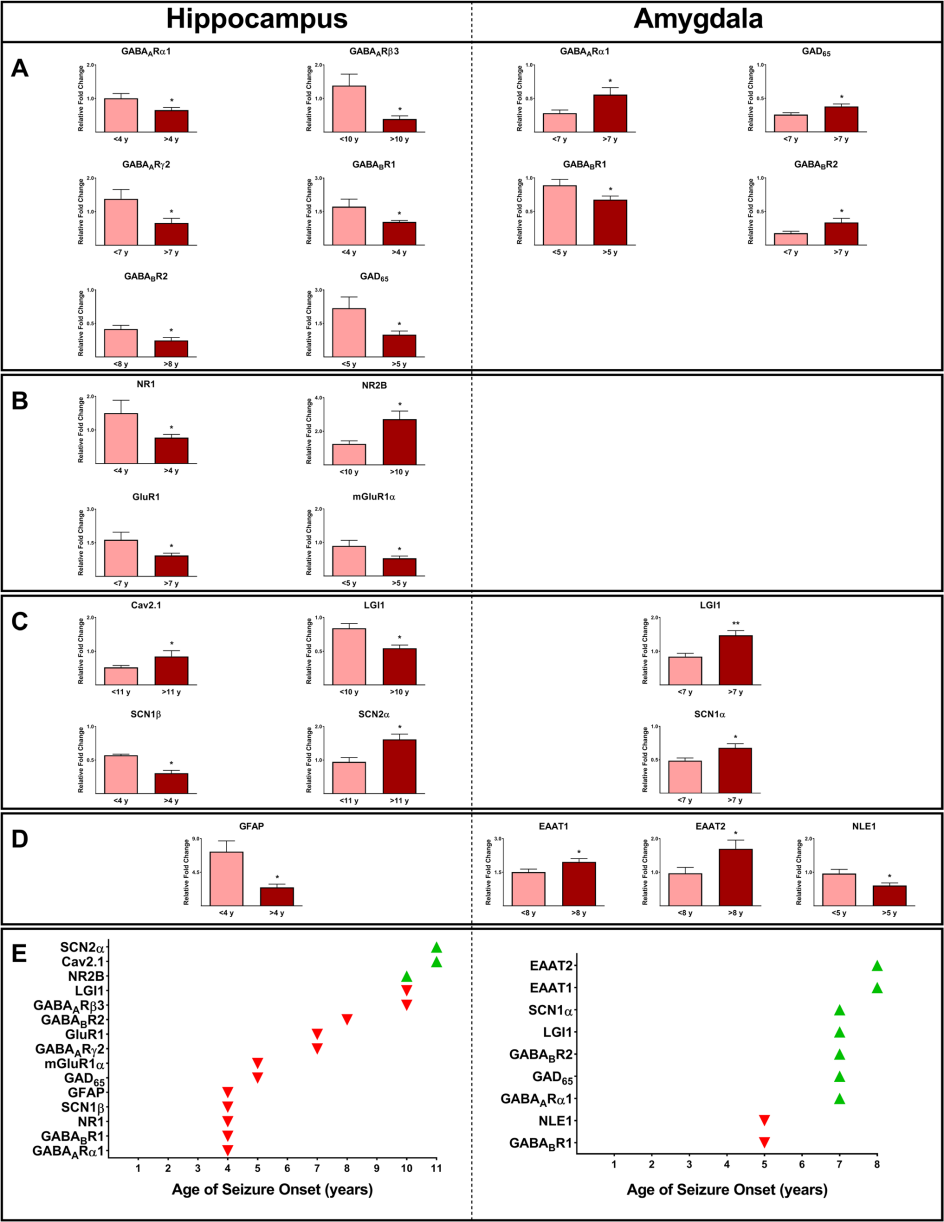
The effect of the age of seizure onset on the expression profile of various genes in the hippocampus and amygdala samples obtained from epileptic patients. Our evaluation revealed distinct gene expression profiles across the age of seizure onset among the patients. Different expression profiles of various GABA receptors and GAD_65_ (**A**), glutamate receptors (**B**), ion channels, and glioma-inactivated 1 gene (LGI1) (**C**), as well as GFAP, NLE1, and EAAT2 (**D**), in the epileptic hippocampus and amygdala were observed. An overview of the relationships between changes in various biomarkers and the age of seizure onset is shown. * and ** indicate *P* ≤ 0.05 and *P* ≤ 0.01, respectively

We analyzed patients’ data to evaluate whether the age of seizure onset had an impact on the expression profile of various genes. The age of seizure onset ranged from 1 to 32 years old (11.6 ± 2 years; Table 1). The mean age of patients at the time of surgery was 33.2 ± 2.6 years (range from 14 to 52 years). Our analyses have shown distinct gene expression profiles across the age of seizure onset among the patients. The expression values of GABA_A_Rα1, GABA_B_R1, NR1, SCN1β, and GFAP were lower in the hippocampus of the patients with an age of seizure onset of >4 years compared to <4 years (*P* ≤ 0.05; Fig. 7A–D). Furthermore, the expressions of GAD_65_ and mGluR1α in the hippocampus of patients with a seizure onset of >5 years were lower than those with an onset of <5 years (*P* ≤ 0.05; Fig. 7A,B). The values of GABA_A_Rγ2 and GluR1 were significantly lower in the hippocampal tissues of patients with an age of seizure onset of >7 years compared with the patients with an age of seizure onset of <7 years (*P* ≤ 0.05; Fig. 7A,B). The expression values of GABA_B_R2 were lower in the hippocampus of the patients with an age of seizure onset of >8 years compared to <8 years (*P* ≤ 0.05; Fig. 7A). Significant lower values of GABA_A_Rβ3 and LGI1 genes, as well as a higher expression of NR2B, were observed in the hippocampus of epileptic patients with an age of seizure onset of >10 years compared to the subjects with an age of onset of <10 years (*P* ≤ 0.05; Fig. 7A–C). Patients with the age of seizure onset of >11 years showed a significantly higher expression of Cav2.1 and SCN2α in the hippocampus compared to those with the age of seizure onset of less than 11 years (*P* ≤ 0.05; Fig. 7C).

In the amygdala, the values of GABA_B_R1 and NLE1 genes were significantly lower in patients with an age of seizure onset of >5 years compared to <5 years (*P* ≤ 0.05; Fig. 7A,D). The expression values of GABA_A_Rα1, GAD_65_, GABA_B_R2, LGI1, and SCN1α were significantly higher in the amygdala of the patients with an age of seizure onset of >7 years compared to <7 years (*P* ≤ 0.05; Fig. 7A,C). The expressions of both EAAT1 and EAAT2 in the epileptic amygdala were significantly higher in patients with an age of seizure onset of >8 years compared to those subjects with an age of seizure onset of <8 years (*P* ≤ 0.05; Fig. 7D). Figure 7E represents an overview of the relationships between alterations of various studied biomarkers and the age of seizure onset.

### Epilepsy-associated Gene Expression and Duration of Epilepsy

**Fig. 8:**
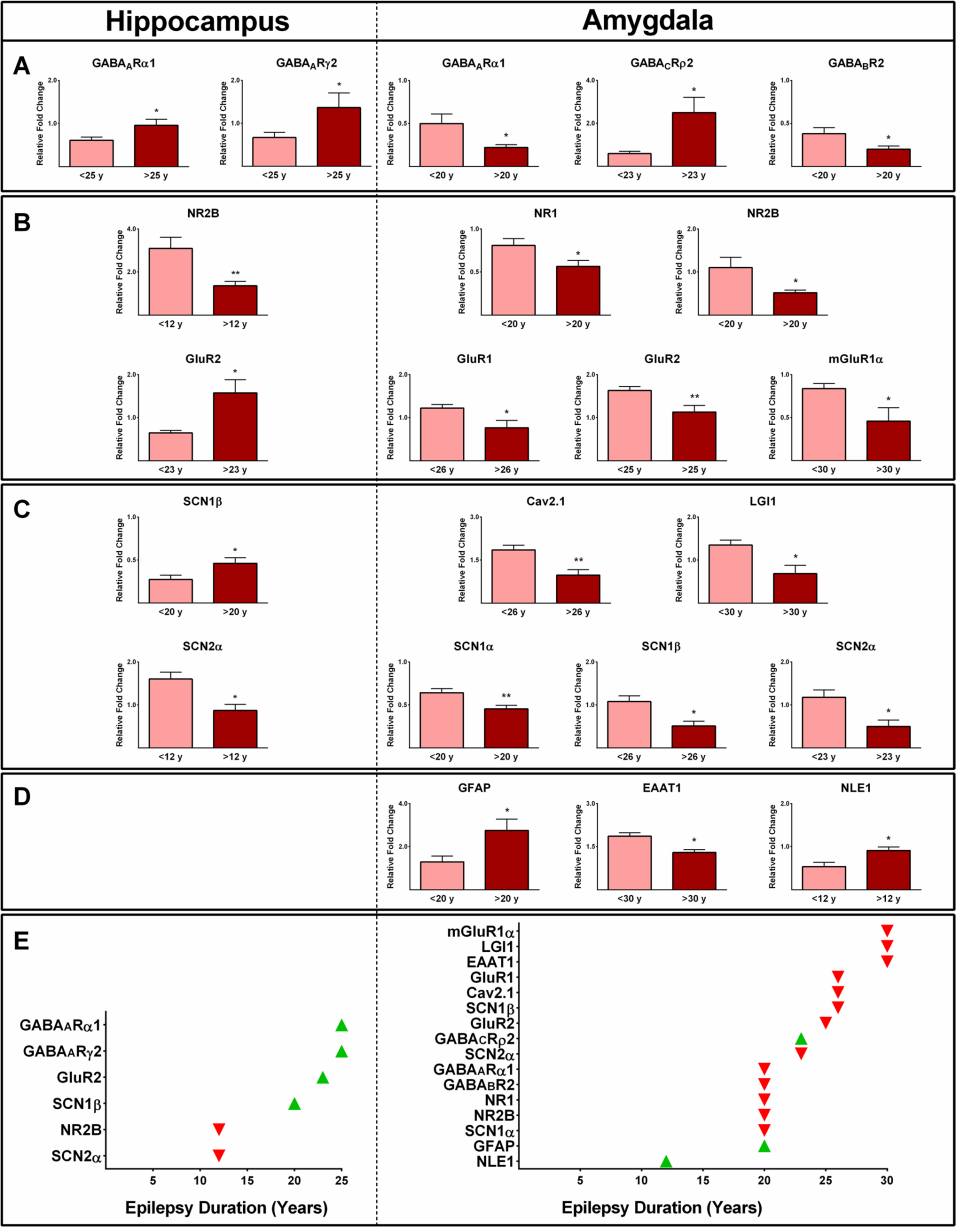
The effect of the duration of epilepsy on the expression profile of various genes in the hippocampus and amygdala samples obtained from epileptic patients. Our study revealed distinct gene expression profiles among the patients with different duration of epilepsy. Different expression profiles of various GABA receptors and GAD_65_ (**A**), glutamate receptors (**B**), ion channels, and glioma-inactivated 1 gene (LGI1) (**C**), as well as GFAP, NLE1, and EAAT1 (D), in the epileptic hippocampus and amygdala were observed. An overview of the relationships between changes in various biomarkers and the duration of epilepsy is shown. * and ** indicate *P* ≤ 0.05 and *P* ≤ 0.01, respectively

We also assessed whether the duration of epilepsy had an influence on the expression profile of the detected genes [37]. Duration of epilepsy in our patients ranged from 3 to 45 years (21.6 ± 2.5 years; Table 1). The expression values of GABA_A_Rα1 and GABA_A_Rγ2 in the hippocampus of the patients with epilepsy duration of >25 years were significantly higher than patients with epilepsy duration of <25 years (*P* ≤ 0.05; Fig. 8A). A higher value of GluR2 expression was observed in the hippocampus of the patients with epilepsy of >23 years compared with those with a duration of <23 years (*P* ≤ 0.05; Fig. 8B). The expression of SCN1β genes was significantly higher in the hippocampus of subjects with epilepsy of >20 years compared with patients with duration of <20 years (*P* ≤ 0.05; Fig. 8C). The expression levels of NR2B and SCN2α genes in the hippocampus were lower in subjects with epilepsy duration of >12 years compared with those patients with duration of <12 years (*P* ≤ 0.05; Fig. 8B,C).

The expression levels of mGluR1α, LGI1, and EAAT1 in the amygdala of the patients with epilepsy duration of >30 years were significantly lower than in subjects with a duration of <30 years (*P* ≤ 0.05; Fig. 8B–D). Moreover, the expressions of GluR1, Cav2.1, and SCN1β were also significantly lower in the amygdala of subjects with epilepsy duration of >26 years than those with a duration of <26 years (*P* ≤ 0.05; Fig. 8B,C). The value of GluR2 gene expression was significantly lower in the amygdala tissues of patients with epilepsy duration of >25 years than in patients with epilepsy duration of <25 years (*P* ≤ 0.05; Fig. 8B). Additionally, in the amygdala of epileptic patients with a duration of epilepsy exceeding 23 years, there was a significant increase in the expression of GABA_C_Rρ2 and a decrease in the expression of SCN2α compared to patients with a duration of epilepsy less than 23 years (*P* ≤ 0.05; Fig. 8A,C). Significantly lower expression levels of GABA_A_Rα1, GABA_B_R2, NR1, NR2B, and SCN1α genes were observed in the amygdala of patients with an epilepsy duration exceeding 20 years compared to those with epilepsy duration less than 20 years (*P* ≤ 0.05; Fig. 8A–D). There was a higher expression of GFAP in the amygdala of patients with epilepsy lasting for more than 20 years compared to those with epilepsy duration of less than 20 years (*P* ≤ 0.05; Fig. 8A–D). The value of NLE1 was significantly higher in the amygdala of patients with epilepsy duration of >12 years compared to those with epilepsy duration less than 12 years (*P* ≤ 0.05; Fig. 8D). Figure 8E provides an overview of the relationships between alterations in various studied genes and the duration of epilepsy.

### Epilepsy-associated Gene Expression and Number of Seizures

**Fig. 9:**
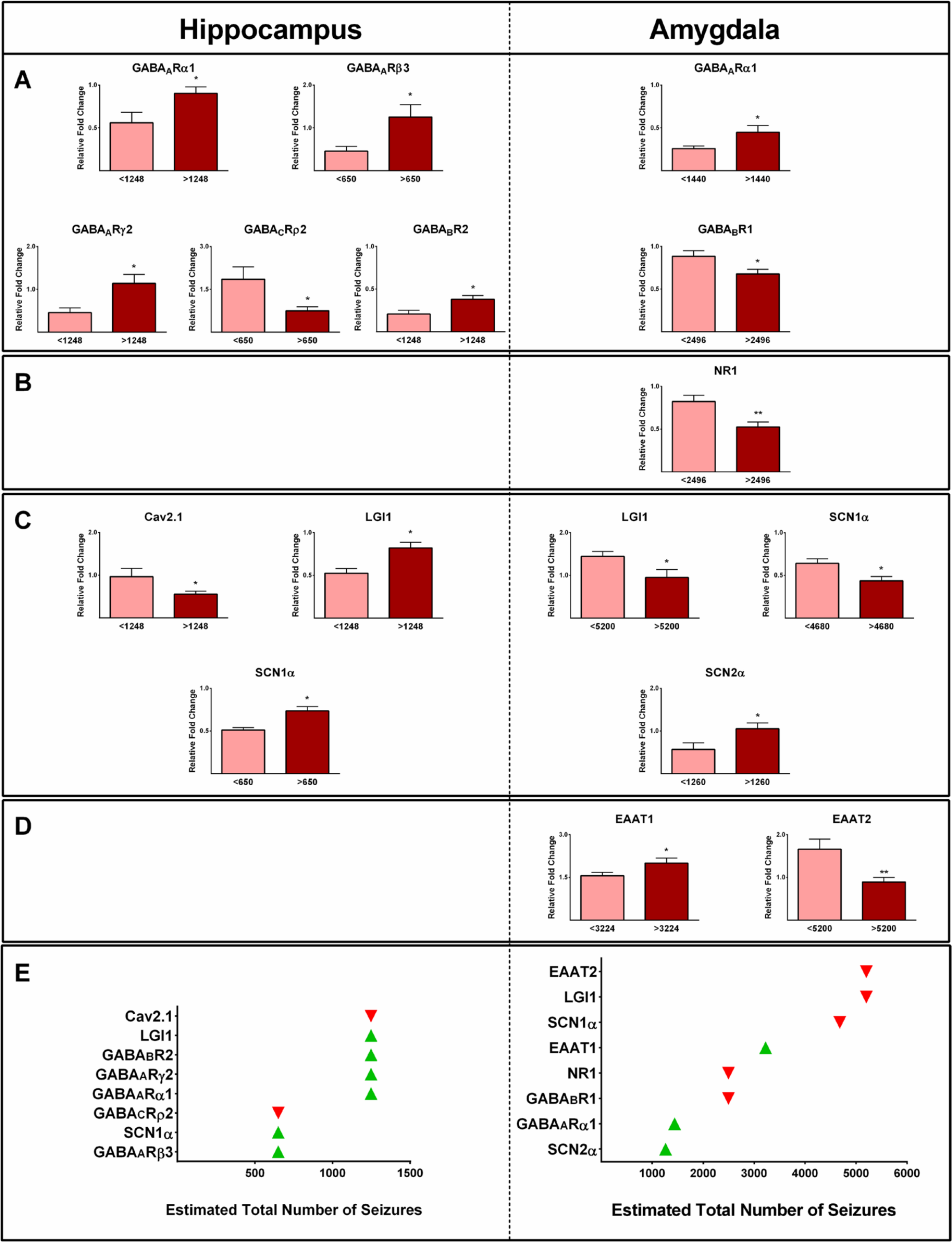
The effect of the estimated total number of seizures on the expression profile of various genes in the hippocampus and amygdala samples obtained from epileptic patients. Our investigations revealed distinct gene expression profiles among the patients with different estimated total numbers of seizures. Different expression profiles of various GABA receptors and GAD65 (**A**), glutamate receptors (**B**), ion channels, and glioma-inactivated 1 gene (LGI1) (**C**), as well as EAAT1 and EAAT2 (**D**), in the epileptic hippocampus and amygdala were observed. An overview of the relationships between changes in various biomarkers and the estimated total number of seizures is shown. * and ** indicate *P* ≤ 0.05 and *P* ≤ 0.01, respectively

In addition to the age of seizure onset and the duration of epilepsy, seizure frequency can also have an impact on the expression of genes in MTLE [38]. To estimate the total number of seizures, we utilized the number of seizures reported by the patients and their respective duration of epilepsy. The estimated total number of seizures experienced by the 19 patients before undergoing surgery ranged from 72 to 116,800 (Table 1). The values of GABA_A_Rβ3 and SCN1α gene expression in the hippocampus were significantly higher in patients with an estimated total number of >650 seizures than in subjects with a total seizure number of <650 (*P* ≤ 0.05; Fig. 9A,C). However, the expression levels of GABA_C_Rρ2 in the hippocampus were significantly lower in subjects with a total number of >650 seizures than those with total seizures of <650 (*P* ≤ 0.05; Fig. 9A). The expression of GABA_A_Rα1, GABA_A_Rγ2, and GABA_B_R2, as well as LGI1, in the hippocampus was significantly higher in patients with a total seizure number of >1248 than in subjects with total seizures of <1248 (*P* ≤ 0.05; Fig. 9A,C). The expression of Cav2.1 in the hippocampus was significantly lower in patients with a total number of seizures of >1248 than in subjects with <1248 (*P* ≤ 0.05; Fig. 9C).

The levels of SCN2α and GABA_A_Rα1 in the amygdala of patients with the estimated total number of seizures of >1260 and >1440 were significantly higher than those with <1260 and <1440, respectively (*P* ≤ 0.05; Fig. 9A,C). The values of GABA_B_R1 and NR1 expressions in the amygdala of subjects with an estimated number of seizures of >2496 were significantly lower than in those with <2496 (*P* ≤ 0.05; Fig. 9A,B). Moreover, the levels of EAAT1 expression in the amygdala were found to be significantly higher in patients with an estimated total number of seizures greater than 3224 compared to those with a total number of seizures less than 3224 (*P* ≤ 0.05; Fig. 9D). The gene expression of SCN1α in the amygdala of patients with a total number of seizures of >4680 was lower than patients with <4680 (*P* ≤ 0.05; Fig. 9C). The values of LGI1 and EATT2 expressions in the amygdala were significantly lower in patients with total seizures of >5200 seizures than those with total seizures of <5200 (*P* ≤ 0.05; Fig. 9C,D). Figure 9E provides an overview of the relationships between changes in various studied biomarkers and the estimated total number of seizures.

### Epilepsy-associated Gene Expression and the Frequency of Seizures

**Fig. 10:**
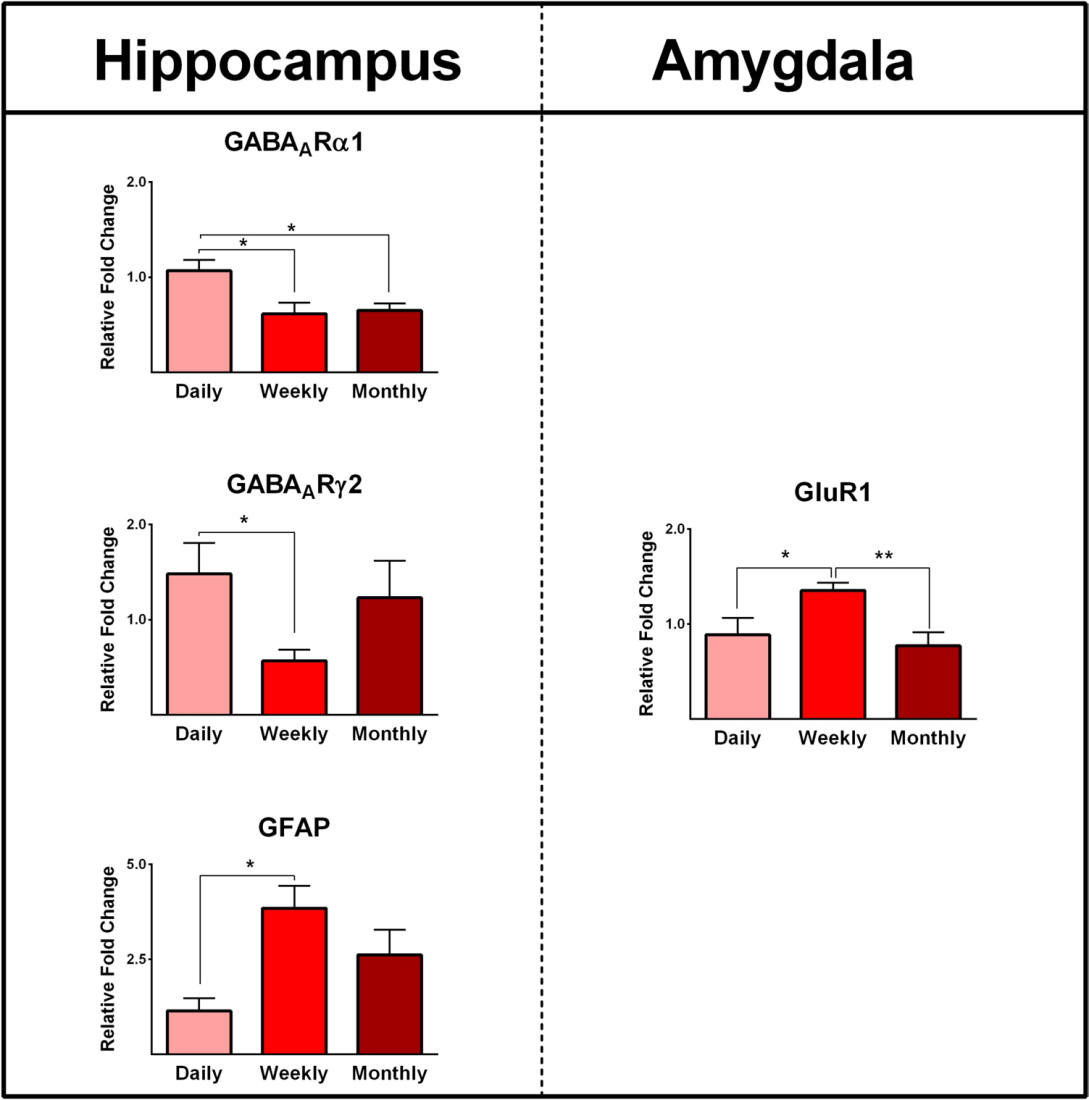
The effect of seizure frequency (daily, weekly, and monthly) on gene expression profiles in the epileptic hippocampus and amygdala. The expression levels of GABAARα1, GABAARγ2, and GluR1 in the epileptic hippocampus and amygdala were significantly different among patients with various seizure frequencies. * and ** indicate *P* ≤ 0.05 and *P* ≤ 0.01, respectively

Seizure frequency was categorized into three groups: daily, weekly, and monthly. We then evaluated the potential effects of seizure frequency on gene expression profiles. The expression of GABA_A_Rα1 in the hippocampus was significantly higher in patients with daily seizures compared to subjects with weekly and monthly seizures (*P* ≤ 0.05; Fig. 10). Furthermore, the value of GABA_A_Rγ2 in the hippocampus was significantly higher in patients with daily seizures compared to subjects with weekly seizures (*P* ≤ 0.05; Fig. 10). The expression of GFAP genes was significantly higher in the hippocampus of patients with weekly seizures compared to those with daily seizures (*P* ≤ 0.05; Fig. 10). Moreover, we observed that the gene expression of GluR1 in the amygdala was significantly higher in patients with weekly seizures compared to those with daily and monthly seizures (*P* ≤ 0.05; Fig. 10).

### Epilepsy-associated Gene Expression and Anticonvulsive Therapy

**Fig. 11:**
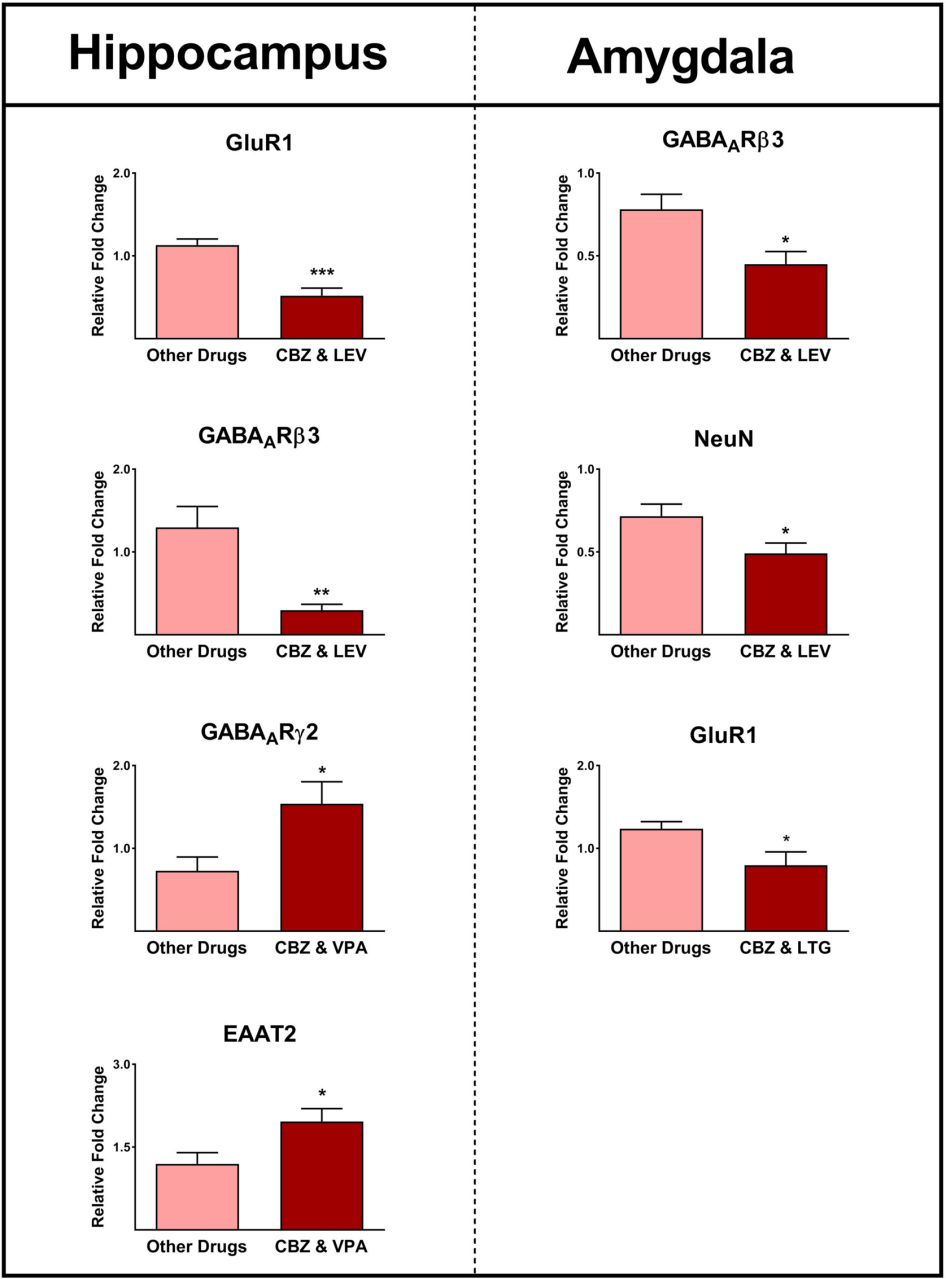
The effect of anticonvulsant therapy on gene expression profiles in the epileptic hippocampus and amygdala. The expression levels of GluR1, GABA_A_Rβ3, GABA_A_Rβ3, GABA_A_Rγ2, NeuN, and EAAT2 in the epileptic hippocampus and amygdala were significantly different among patients using various anticonvulsants. *, **, and *** indicate *P* ≤ 0.05, *P* ≤ 0.01, and *P* ≤ 0.001, respectively

The most frequently prescribed AEDs in our patients included carbamazepine, valproic acid, levetiracetam, and lamotrigine (Table 1). We conducted an evaluation to investigate the effects of commonly prescribed AEDs on the gene expression profiles of our patients. In the hippocampus, we observed significantly lower expressions of GluR1 and GABA_A_Rβ3 genes in patients who were treated with carbamazepine and levetiracetam compared to patients treated with other AEDs (*P* ≤ 0.001; Fig. 11). In the hippocampus, the expressions of GABA_A_Rγ2 and EAAT2 genes were significantly higher in patients who were treated with carbamazepine and valproic acid compared to those who received other AEDs (*P* ≤ 0.05; Fig. 11). Furthermore, we observed significantly lower expressions of GABA_A_Rβ3 and NeuN genes in the amygdala of patients who were treated with carbamazepine and levetiracetam compared to those who received other AEDs (*P* ≤ 0.05; Fig. 11). In the amygdala, we found significantly lower expressions of GluR1 gene in patients who were treated with carbamazepine and lamotrigine compared to those who received other drugs (*P* ≤ 0.05; Fig. 11).

### Epilepsy-associated Gene Expression and Type of Seizures

**Fig. 12:**
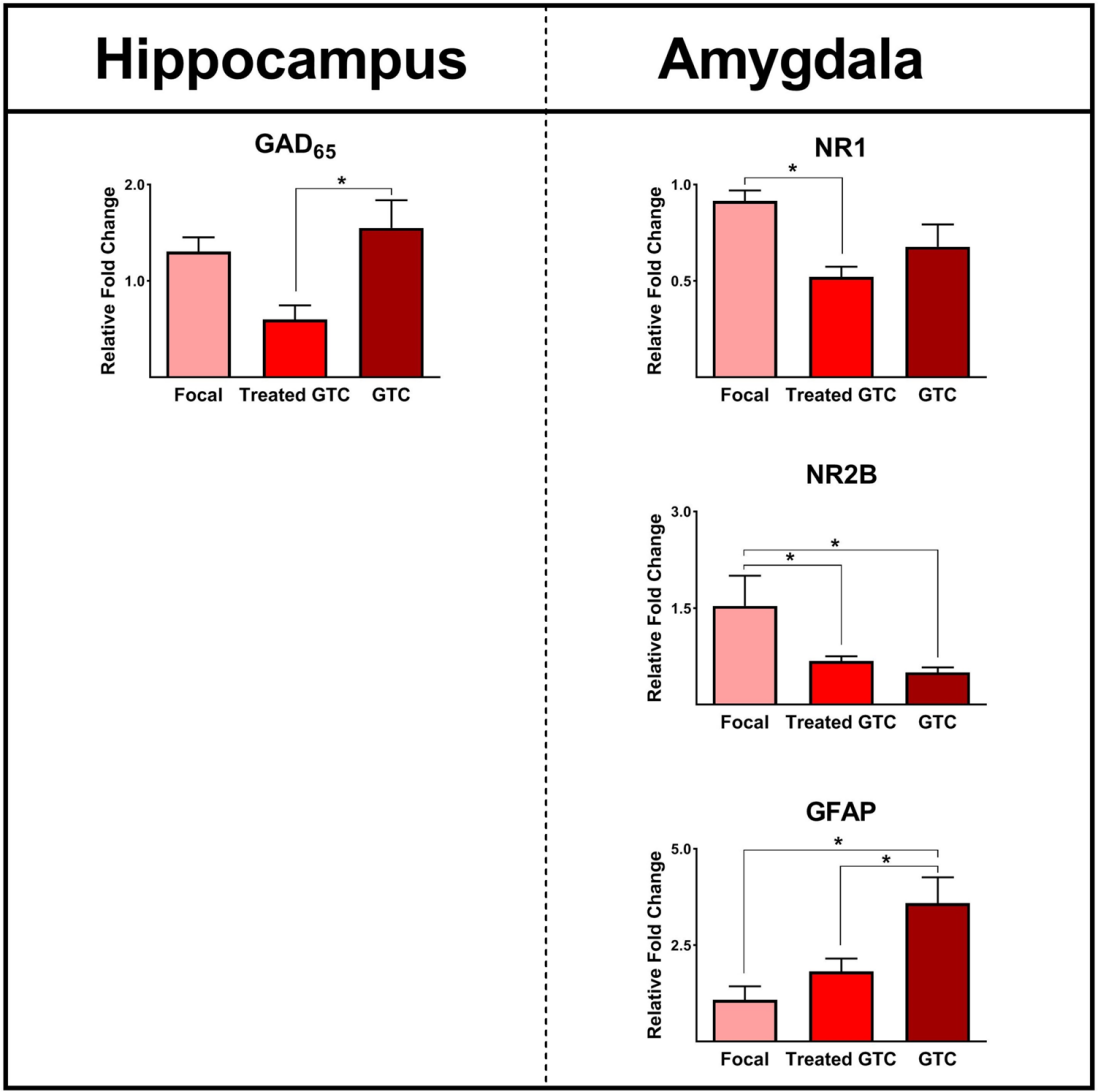
Various types of seizures (focal and treated and non-treated secondarily generalized tonic-clonic seizures) affect gene expression profiles in the epileptic hippocampus and amygdala. The expression levels of GAD65, NR1, NR2B, and GFAP in the epileptic hippocampus and amygdala were significantly different among patients with varying types of seizures. * indicates *P* ≤ 0.05

The seizures were categorized as focal seizures (only focal seizures without generalization), generalized tonic colonic seizures (GTC, secondarily generalized tonic-clonic seizures with or without preceding focal seizure in spite of drug treatment), and treated GTC (history of GTC, but secondarily generalized seizures controlled by AEDs). The possible effects of these seizure types on gene expression profiles were evaluated. Patients with GTC have a significantly higher expression of GAD_65_ in the hippocampus compared with patients with treated GTC (*P* ≤ 0.05, Fig. 12). Furthermore, the expression levels of NR1 in the amygdala of treated GTC patients were significantly lower than in patients with focal seizures (*P* ≤ 0.05, Fig. 12). A significant enhancement of NR2B expression in the amygdala was observed in patients experiencing focal seizures, distinguishing them from other groups. Moreover, the expression of GFAP was significantly elevated in the group with GTC, presenting a marked contrast to the remaining groups (*P* ≤ 0.05, Fig. 12).

### Epilepsy-associated Gene Expression and Dominant Lobe

**Fig. 13:**
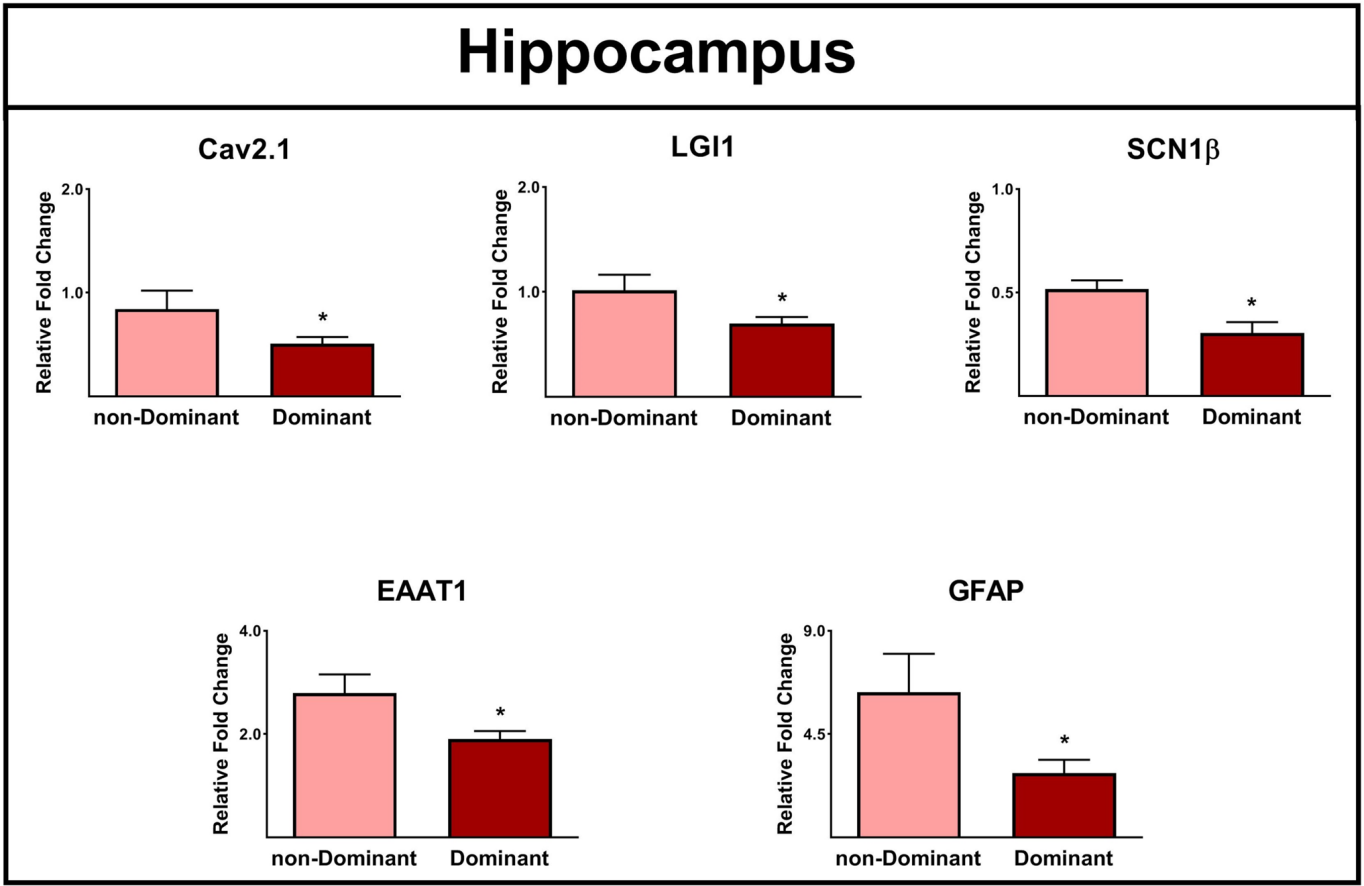
The effect of interhemispheric gene expression (dominant vs. non-dominant lobe) on gene expression profiles in the epileptic hippocampus. The expression levels of Cav2.1, LGI1, SCN1β, EAAT1, and GFAP in the epileptic hippocampus were significantly different among patients with the epileptic zone in the dominant vs. non-dominant lobe. * indicates *P* ≤ 0.05

It has been suggested that varying interhemispheric gene expression within the temporal cortex of humans contributes to hemispheric lateralization [39]. In our study, significantly reduced expressions of Cav2.1, LGI1, SCN1β, EAAT1, and GFAP genes were observed in the hippocampus of patients with an epileptogenic zone in the dominant lobe compared to those who underwent resection in the non-dominant lobe (*P* ≤ 0.05, Fig. 13). No significant differences were observed in gene expression of different biomarkers between the amygdala resected from dominant and non-dominant hemispheres.

### Epilepsy-associated Gene Expression and Gender

**Fig. 14:**
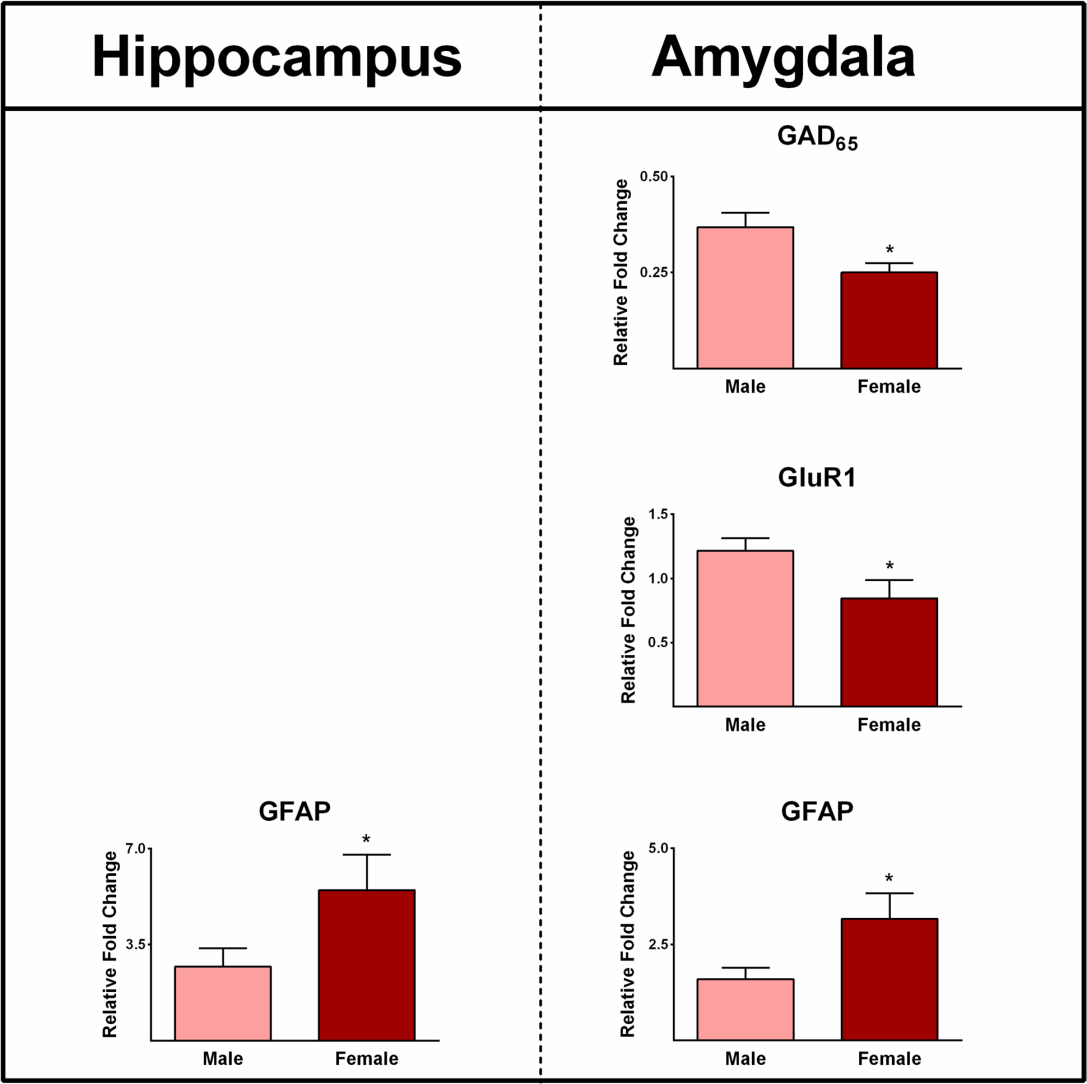
The differences in gene expression profiles between the epileptic hippocampus and amygdala in different genders. The levels of GFAP expression in the epileptic hippocampus and amygdala were found to be significantly higher in female epileptic patients compared to males. Additionally, the expression levels of GAD_65_ and GluR1 in the amygdala were significantly lower in females with medically refractory epilepsy when compared to males. * indicates *P* ≤ 0.05

Consideration of gender issues is crucial in understanding and effectively managing epilepsy [40]. In our study, we observed significant differences in the expression of GFAP genes in the hippocampus and amygdala between female and male patients (*P* ≤ 0.05, Fig. 14). Furthermore, the expression levels of GAD_65_ and GluR1 in the amygdala were significantly lower in female patients compared to male patients (*P* ≤ 0.05, Fig. 14).

### Epilepsy-associated Genes as Predictors for the Clinically Relevant Variables

Subsequently, we investigated whether the expression levels of any of the examined markers could serve as predictors for clinically relevant variables such as the total number of seizures, duration of epilepsy, and age of seizure onset. As described in the “Materials and Methods” section, we performed multiple linear regression using gene expression values as predictors for different variables. Among different biomarkers, the expression of NR1 in the hippocampus had the greatest association with the estimated total number of seizures. Furthermore, the expression of SCN1β and NeuN in the hippocampus exhibited the strongest association with the duration of epilepsy. Moreover, multiple regression analysis predicted that the expression of SCN1α in the hippocampus had the most considerable association with the age of seizure onset.

In the amygdala, multiple regression analyses showed that the expression of GluR2 and Cav2.1, as well as GFAP and EAAT1, could best predict the total number of seizures and epilepsy duration, respectively. Moreover, our analyses demonstrated that the expression of EAAT1, GAD_65_, and GABA_A_Rβ3 were the best predictors of the age of seizure onset (Table 4).

**Table 4:** Analyses of multiple regression between all markers in the hippocampus and amygdala tissue

Hippocampus	Estimated Total Number of Attacks					
	Models	Markers	Β-Coefficients	Variable *P* Value	Model *P* Value	Adjust R Square
	1	NR1	0.95	0.002	0.002	0.90
	Epilepsy Duration					
	1	SCN1β	0.81	0.048	0.048	0.58
	2	SCN1β	1.4	0.009	0.02	0.88
		NeuN	0.78	0.045		
	Age of Seizure Onset					
	1	SCN1α	0.95	0.003	0.003	0.89
Amygdala	Estimated Total Number of Attacks					
	1	GluR2	0.73	0.04	0.04	0.45
	2	GluR2	1.21	0.003	0.007	0.81
		Cav2.1	-0.75	0.02		
	Eüilepsy Duration					
	1	GFAP	0.79	0.02	0.02	0.57
	2	GFAP	0.79	0.004	0.005	0.84
		EAAT1	0.50	0.02		
	Age of Seizure Onset					
	1	EAAT1	0.83	0.01	0.01	0.64
	2	EAAT1	0.81	0.003	0.004	0.85
		GAD_65_	0.45	0.03		
	3	EAAT1	0.79	0.001	0.002	0.95
		GAD_65_	0.58	0.004		
		GABA_A_Rβ3	-0.31	0.03		

## Discussion

Epilepsy is a multifaceted disorder characterized by profound disruptions in various mechanisms involved in the regulation of receptor functions, ion homeostasis, energy metabolism, and/or transmitter uptake within the brain. The altered expression of a diverse genes represents one of the primary changes that occur during epileptogenesis. Determining the alterations in gene expression as causative and/or consequential factors in the development of seizures is crucial to establishing novel preventive and therapeutic approaches. Our findings highlight large-scale and complex alterations in the values of several key regulatory genes in both the hippocampus and amygdala of patients with medically intractable MTLE. The changes in the expressions of various genes differed significantly between hippocampal and amygdala samples. Distinct correlation patterns were observed between alterations in gene expression and clinical characteristics, depending on whether the epileptic patients were considered as a unified group or subdivided into different groups (Table 5).

**Table 5:**
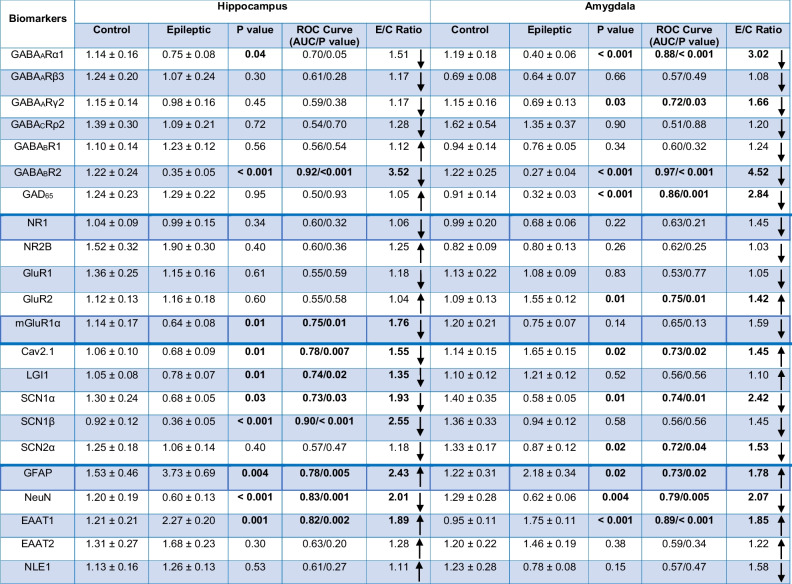
Relative gene expression levels in epileptic and control groups

### Alterations of Various Gene Expressions in Epileptic Specimens

Recent evidence regarding the role of the excitation/inhibition imbalance in seizure development suggests that epileptogenesis is influenced not only by changes in receptors and ion channels but also by genetic and metabolic factors, neurotransmitters, and the effects of AEDs, which collectively contribute to the process of epileptogenesis [41]. Our findings frequently in line with those of previous investigations. Distinct alterations in the expression of inhibitory receptors and enzymes have been observed in the epileptic hippocampus and amygdala. While the expression levels of the GABA_A_Rα1 and GABA_B_R2 were lower in the hippocampus and amygdala, the reduction of GABA_A_Rγ2 and GAD_65_ has been observed only in the amygdala. Impaired functions of GABA_A_Rα1 [42, 43], GABA_A_Rγ2 [44], GABA_B_R2 [45], and GAD_65_ [46, 47] in the amygdala lead to brain hyperexcitability and seizures [48]. Furthermore, the reduction of GABA_A_Rα1 and GAD_65_ expression was associated with a higher rate of cell damage and apoptosis in the human epileptic amygdala [11]. Our study revealed significant alterations of mGluR1α expression in the hippocampus and GluR2 in the epileptic amygdala. Upregulation of mGluR1α in the hippocampus of patients with MTLE [49, 50] and dysfunction of GluR2 in the amygdala [51] contribute to brain hyperexcitability and epilepsy. Epileptic seizures lead to the downregulation of GluR2 mRNA and subunit expression in the hippocampal pyramidal cells [52]. Moreover, our findings illustrated the significant changes in Cav2.1, SCN1α, SCN1β, SCN2α, and LGI1 in the epileptic hippocampus and amygdala compared with controls. Changes in the CaV2.1 subunit of the P/Q-type Ca^2+^ channel [53], NaV1.1 channel encoded by the SCN1A gene [54], as well as other subunits of sodium channels [55, 56], and LGI1 [57, 58], have a key role in the regulation of neuronal network excitability and seizures. In keeping with our findings, significant alterations of EAAT1 and GFAP have been observed in the hippocampus and temporal lobe of epileptic patients with hippocampal sclerosis [23, 59, 60]. The reduction of EAAT could be part of the underlying mechanism of impaired glutamate clearance and neuronal hyperexcitability in epilepsy [61, 62]. The changes in GFAP and NeuN expressions in our study can be related to neuronal cell loss with concomitant astrogliosis in MTLE specimens [63].

### Epilepsy-associated Gene Expression and Clinical Characteristics

Our data revealed correlations between the altered expression of various genes with the age of patients, the age of seizure onset, epilepsy duration, and the number of seizures. Altered expression of microRNA (miRNAs) in the human brain plays a key role in the age of seizure onset [64], and dysregulated miRNAs can target both the excitatory and inhibitory receptors, as well as different ion channels, which may lead to neuronal excitability and seizures [37]. Mutations and changes in the expression of genes encoding GABA_A_Rγ2 and GABA_A_Rα1 contribute to the age of seizure onset in various epilepsy syndromes [65, 66]. Consistent with our findings, a previous study demonstrated a positive correlation between the duration of illness and the alteration of benzodiazepine receptor ligand binding in temporal lobe specimens resected from patients with intractable epilepsy [67]. Moreover, disparities in the transcript abundance of a number of genes were observed in patients with MTLE exhibiting different seizure frequencies. These findings suggest that a higher seizure frequency is associated with a greater altered signaling pathways that regulate neuronal and synaptic excitability [68]. The variations in the expression of inhibitory receptors, such as the GABARα1 subunit, among certain patients may serve as an adaptive mechanism to reduce the frequency or intensity of epilepsy [69].

One of the major challenges in epilepsy research is the broad clinical heterogeneity of the illness, such as differences in seizure duration and severity, comorbidities, age of onset, and risk factors that should be considered in developing personalized and safer therapies [70, 71]. A great variability has been observed in the seizure network evolutions in human epileptic brain tissues [72], which could arise from various modulatory processes and result in different seizure patterns in each patient [73]. We found different correlation patterns between the alterations of gene expression and clinical characteristics when the epileptic cohort was subdivided. Differential neuropathological alterations and receptor expression in MTLE with and without psychiatric comorbidities suggest that psychological states rely on various morphological and neurochemical states [74]. In keeping with our findings, previous investigations have demonstrated that clinical factors such as seizure frequency and epilepsy duration could significantly contribute to disruptions in the GABAergic system, including GABA_A_Rα1-6, GABA_A_Rα1-6, GABA_A_Rγ, and GABA_B_ receptors, in patients with MTLE and psychiatric comorbidities [75]. We found significant alterations in the expression of different voltage-gated sodium channels and LGI1 in both the amygdala and hippocampus of MTLE patients with psychological comorbidities. It has been shown that mutations in the SCN1α [76], SCN2α [77], and LGI1 [78] genes are responsible for different epilepsy disorders and associated neuropsychiatric abnormalities.

AEDs could modulate the expression of various receptors. Increased brain GABA levels following a long-term administration of some AEDs, such as gabapentin, tiagabine, and vigabatrin, could lead to changes in the function of both GABA_A_R and GABA_B_R [79–83]. AEDs with mood-stabilizing properties, such as valproate acid and carbamazepine, enhance the number of GABA_B_R-binding sites in the rat hippocampus [84]. Certain AEDs, like valproate and gabapentin, enhance the GABA and GAD turnover in a regionally selective manner [85, 86]. The glutamate receptor alterations, particularly AMPA synaptic reorganization, mediate the brain excitability both prior to the occurrence of seizures and when seizures develop [25]. Anticonvulsant effects of some AMPA antagonists, such as perampanel, indicate the key role of these receptors in seizure generation and propagation [87]. Furthermore, the adverse effects of specific AEDs can be linked to the occurrence of psychotic disorders in epileptic patients, potentially through the modulation of various receptor signatures [88, 89].

Gender differences play a significant role in epilepsy, exerting notable influences on different aspects of the disease. These differences are likely due to the modulatory effects of steroid hormones and endogenous neurosteroids on synaptic transmission, as well as the function of neurons and astrocytes [90]. Both sex and gender factors have implications not only for diagnostic assessments but also for therapeutic choices [40, 91]. In accordance with our findings, previous experimental studies have demonstrated that female rats exhibit a significantly higher number of GFAP-positive cells and increased astrogliosis in the hippocampus following spontaneous recurrent seizures in comparison to males [92]. Our findings revealed a significant decrease in GAD_65_ expression within the amygdala of female epileptic patients. Existing evidence suggests that estradiol has the potential to decrease the seizure threshold and suppress the activity of GAD in the amygdala [93].

The functional integration of the temporal lobe relies on complex reciprocal neural network connections between the hippocampus and amygdala. During epileptogenesis, the dysfunction of these structures profoundly impacts the bidirectional inter-regional information flow, leading to diverse alterations in neuronal activity, synaptic plasticity, as well as receptor function and distribution [94, 95]. Transcriptomic analysis carried out in rats subjected to hippocampal and amygdaloid kindling revealed shared and distinct complex alterations in gene expression within both the hippocampus and amygdala. These alterations included changes in genes encoding glutamate and GABA receptors, various ion channels, neurogenesis-related genes, and inflammatory genes [96]. In our study, the epileptic hippocampus and amygdala exhibited distinct alterations in several molecular signatures. The roles and contributions of the amygdala and hippocampus in medically refractory epilepsy can vary significantly, depending on the underlying pathology, the location of the epileptic focus, and individual differences in neuronal circuitry [97]. Variations in ion channel and receptor density, along with their regulation under physiological and pathophysiological conditions, have been observed between the hippocampus and amygdala [18, 98]. These differences give rise to distinct regulatory mechanisms in neuronal excitability within these brain structures [99]. Numerous studies conducted on animal and human epileptic tissues have unveiled distinct expression patterns of various GABA subreceptors [99, 100] and glutamate subreceptors [97, 101, 102] within the hippocampus and amygdala. In patients with MTLE, gene alterations attributed to epigenetic factors reveal distinct methylation patterns in genes responsible for voltage-gated channels, neurotransmitter receptors, and neuroinflammatory cascades within the hippocampus and amygdala. These genes participate in signaling pathways known to be associated with MTLE, including PKC activation through G-Proteins, Trk, and p75NTR neurotrophic pathways [103]. Further studies are warranted to determine the relevance and importance of differential or even inverse gene expression in the hippocampus and amygdala in patients with MTLE.

Examining the expression, localization, and activity of various receptors shows promise in identifying potential biomarkers for epilepsy. Engel et al. [104] suggested alterations in GABA receptors or sodium channels as biomarkers, but validation through human epileptic brain studies is needed. Other studies evaluated resected human brain samples to detect specific biomarkers in the epileptic zone [105, 106]. Investigations indicate that GABA receptors, GABA/glutamate/glutamine ratio, glutamate, and various inflammatory mediators may serve as biomarkers for medically refractory epilepsy [106, 107]. Our findings represent the hint that factors differently affected in various groups of patients defined within a given category could be viewed as diagnostic biomarkers. Receptors or channels that distinguish these groups from each other seem to be associated with specific functional consequences of epilepsy. An encapsulation of the overwhelming amount of data presented and the attempt to relate the measured findings to the actual situations of the patients renders an unimaginable complexity of the epileptic process proper. From this complexity, a hypercomplexity emerges when time is taken into account. With every seizure, with every newly administered antiepileptic drug, with every development and manipulation, the interdependence of the epileptogenic factors involved changes. From the clinical point of view that may indicate that a fixed diagnosis, as well as therapy, is inadvisable in the end.

## Data Availability

The data presented in this study are available upon request from the corresponding author.
